# Histone H3K27 acetylation precedes active transcription during zebrafish zygotic genome activation as revealed by live-cell analysis

**DOI:** 10.1242/dev.179127

**Published:** 2019-10-01

**Authors:** Yuko Sato, Lennart Hilbert, Haruka Oda, Yinan Wan, John M. Heddleston, Teng-Leong Chew, Vasily Zaburdaev, Philipp Keller, Timothee Lionnet, Nadine Vastenhouw, Hiroshi Kimura

**Affiliations:** 1 Cell Biology Center, Institute of Innovative Research, Tokyo Institute of Technology, Yokohama 226-8503, Japan; 2 Center for Systems Biology Dresden, Dresden 01307, Germany; 3 Max Planck Institute for the Physics of Complex Systems, Dresden 01187, Germany; 4 Max Planck Institute of Molecular Cell Biology and Genetics, Dresden 01307, Germany; 5 Howard Hughes Medical Institute, Janelia Research Campus, VA 20147, USA; 6 Advanced Imaging Center, Howard Hughes Medical Institute, Janelia Research Campus, VA 20147, USA; 7 Institute for Systems Genetics and Department of Cell Biology, New York University Langone Health, NY 10016, USA

**Keywords:** Chromatin regulation, Histone modifications, Live-cell imaging, Zygotic genome activation

## Abstract

Histone post-translational modifications are key gene expression regulators, but their rapid dynamics during development remain difficult to capture. We applied a Fab-based live endogenous modification labeling technique to monitor the changes in histone modification levels during zygotic genome activation (ZGA) in living zebrafish embryos. Among various histone modifications, H3 Lys27 acetylation (H3K27ac) exhibited most drastic changes, accumulating in two nuclear foci in the 64- to 1k-cell-stage embryos. The elongating form of RNA polymerase II, which is phosphorylated at Ser2 in heptad repeats within the C-terminal domain (RNAP2 Ser2ph), and miR-430 transcripts were also concentrated in foci closely associated with H3K27ac. When treated with α-amanitin to inhibit transcription or JQ-1 to inhibit binding of acetyl-reader proteins, H3K27ac foci still appeared but RNAP2 Ser2ph and miR-430 morpholino were not concentrated in foci, suggesting that H3K27ac precedes active transcription during ZGA. We anticipate that the method presented here could be applied to a variety of developmental processes in any model and non-model organisms.

## Abstract (precis)

**Summary:** FabLEM, an endogenous labeling technique that uses modification-specific antigen-binding fragments, is used to examine changes in histone modification levels and transcription during zygotic genome activation in live zebrafish embryos.

## INTRODUCTION

FabLEM (Fab-based live endogenous modification labeling) is a technique to visualize transcription and histone modification dynamics using modification-specific antigen-binding fragments (Fabs) (Hayashi-Takanaka et al., 2011; Kimura et al., 2015). With FabLEM, dynamic changes in endogenous protein modification levels throughout living cells and embryos can be monitored in real time, without affecting the cell cycle or developmental processes (Hayashi-Takanaka et al., 2009; Hayashi-Takanaka et al., 2011; Yuan and O'Farrell, 2016; Ruppert et al., 2018; Seller et al., 2019). The specificity of Fabs can furthermore be verified in living cells by inhibitor treatments and/or genetic knockout experiments (Hayashi-Takanaka et al., 2011). The nuclear concentration of fluorescent Fabs reflects global modification levels in the nucleus because Fabs transiently bind to the target modification and freely pass through nuclear pores due to their small size. Therefore, relative changes in target modification levels in single cells can be monitored by changes in the nuclear to cytoplasmic intensity (N/C) ratio of a given Fab fluorescence (Hayashi-Takanaka et al., 2011). Thanks to the signal amplification provided by artificial tandem gene arrays, FabLEM has provided ways to monitor live-cell kinetics of glucocorticoid-stimulated transcription activation in cultured cells (Stasevich et al., 2014a). The presence of H3 Lys9 dimethylation (H3K9me2) on heterochromatinized repeats in living *Drosophila* embryos has also been investigated with FabLEM (Yuan et al., 2016).

In this study, we used FabLEM technology to capture the dynamics of histone modifications and the transcription machinery at a defined endogenous locus in live developing embryos. As the modification sites of histones and RNA polymerase II (RNAP2) are conserved throughout animals, we used modification-specific antibodies, the specificity of which was validated in different models, including human and mouse (Hayashi-Takanaka et al., 2011, 2015; Kimura et al., 2008; Stasevich et al., 2014a), chicken (Hori et al., 2014), fly (Ushijima et al., 2012) and nematode (Egelhofer et al., 2011; Ho et al., 2014; Rechtsteiner et al., 2010).

As a proof of principle, we chose a hallmark of epigenetic changes during development, the maternal-to-zygotic transition. In zebrafish, the zygotic genome is silenced for the first several cell cycles, after which transcription is activated (Kane and Kimmel, 1993). How this zygotic genome activation (ZGA) is regulated has been investigated for decades (Schulz and Harrison, 2019; Vastenhouw et al., 2019). Recent analyses have shown that ZGA is not a sudden event. Before the broad onset of transcription (the major ZGA) at the 1k-cell stage, weak transcription activities and transcripts were also detected as early as the 64-cell stage (Chan et al., 2019; Hadzhiev et al., 2019), which is called the minor ZGA. Because of the dynamic nature of post-translational modifications of histone proteins, the modifications are thought to play important roles in regulating transcription activation. ChIP-seq analysis using early zebrafish embryos revealed an enrichment of H3 Lys4 trimethylation (H3K4me3) in promoters of developmentally regulated genes (Lindeman et al., 2010; Vastenhouw et al., 2010). It has been suggested that occupancy of those genes by H3K4me3 prior to ZGA pre-patterns developmental gene expression (Lindeman et al., 2011). A recent report has also shown dynamic changes of H3 Lys27 acetylation (H3K27ac) at promoters and enhancers before and after zebrafish ZGA by ChIP-seq (Zhang et al., 2018). However, the spatiotemporal dynamics of transcription and histone modifications remain unclear. Here, we demonstrate that FabLEM can be applied to living zebrafish embryos in order to reveal changes in histone modifications and active transcription during ZGA at defined loci.

## RESULTS AND DISCUSSION

### Monitoring the major ZGA

**Fig. 1.**
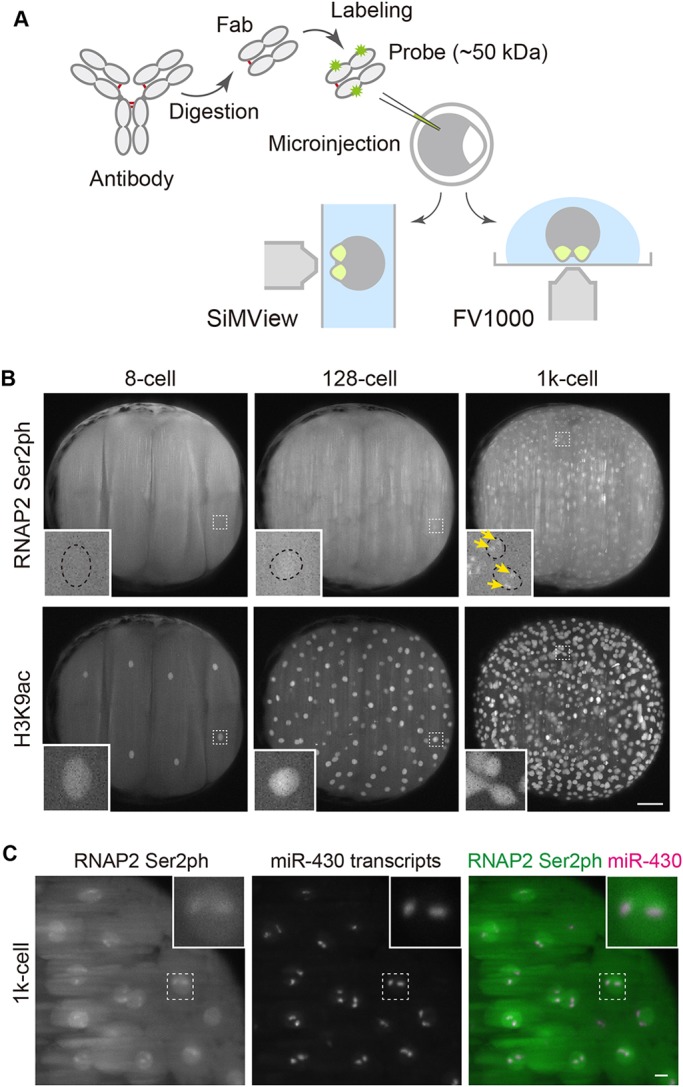
**Visualizing RNA polymerase II and histone modifications in living embryos.** (A) Scheme of experiments. Fluorescently labeled Fabs prepared from modification-specific antibodies are injected into 1-cell-stage zebrafish embryos. After the removal of chorions, the embryos are mounted for a light-sheet (SiMView) or a confocal (FV1000) microscope using low-gelling temperature agarose at the 4-cell stage. (B,C) Representative images taken with a SiMView microscope. (B) Fabs specific to RNAP2 Ser2ph (Alexa 488) and H3K9ac (Cy5) were simultaneously injected and imaged using SiMView. RNAP2 Ser2ph Fabs were clearly concentrated in nuclei around the 1k-cell stage, whereas H3K9ac Fabs were enriched in nuclei from the 8-cell stage. Maximum intensity projections of 198 *z*-sections with 2 μm intervals are shown. Insets show magnified views of the indicated areas. Yellow arrows indicate RNAP2 Ser2ph foci in nuclei. See also Movie 1. Scale bar: 100 μm. (C) Close association of RNAP2 Ser2ph foci with miR-430 transcripts. Embryos were injected with RNAP2 Ser2ph-Fab (Alexa 488) and miR-430 morpholino (Cy3). Maximum intensity projections (30 *z*-planes with 2 μm intervals) at the 1k-cell stage are shown. See also Movie 2. Scale bar: 10 μm.

To monitor transcription activity, we used Fabs specific for the elongating form of RNAP2, which is phosphorylated at Ser2 in heptad repeats within the C-terminal domain (RNAP2 Ser2ph) (Zaborowska et al., 2016). Fluorescently labeled Fabs specific for histone modifications and RNAP2 Ser2ph were injected into 1-cell-stage embryos (Fig. 1A). At the 4-cell stage, embryos were mounted onto a light-sheet (SiMView) (Royer et al., 2016) or a confocal microscope (FV1000), and fluorescence images were collected from the 8- or 16-cell stage up to 6 h post-fertilization (hpf), during which time ZGA occurs. We first examined whether the embryos injected with Fabs develop normally, as do mouse embryos (Hayashi-Takanaka et al., 2009; Hayashi-Takanaka et al., 2011). Embryos injected with Fabs exhibited normal morphology during development, similar to controls without injection or injected with buffer alone (Fig. S1). These data suggest that Fabs injected under the conditions used in this study do not affect early development.

When whole embryos were visualized using a SiMView light-sheet microscope, Alexa 488-labeled RNAP2 Ser2ph-Fabs were uniformly distributed throughout cells at the 8-cell stage with a lack of nuclear foci, consistent with a lack of transcription at this time point (Fig. 1B, Movie 1). In contrast, Cy5-labeled Fabs specific for euchromatic histones acetylated at H3 Lys9 (H3K9ac) were highly enriched in nuclei and mitotic chromosomes. As development continued, however, changes in distribution of RNAP2 Ser2ph-Fabs were observed. Around the 128-cell stage, RNAP2 Ser2ph-Fabs began to accumulate in the nuclei of a few cells, and by the 1k-cell stage nearly all nuclei had strong enrichment. As this timing coincided with the major ZGA, nuclear accumulation of RNAP2 Ser2ph-Fabs appeared to serve as an indicator of transcription activity in developing embryos.

Close inspection of the distribution of RNAP2 Ser2ph-Fabs in the latter stages revealed they were concentrated in two foci (Fig. 1B, inset). These foci were likely to contain alleles of miR-430 gene clusters on chromosome 4 (Chan et al., 2019; Giraldez et al., 2006; Hadzhiev et al., 2019; Heyn et al., 2014; Hilbert et al., 2018preprint). miR-430 family genes play a role in the clearance of maternal RNAs and are therefore expressed at the highest of levels among the early transcribed genes (Hadzhiev et al., 2019; Heyn et al., 2014). miR-430 transcripts have furthermore been visualized using a specific antisense morpholino oligonucleotide (Hadzhiev et al., 2019). To test whether the RNAP2 Ser2ph foci were in fact marking miR-430 transcript clusters, we injected a Cy3-labeled miR-430-specific morpholino (Hadzhiev et al., 2019) with Alexa 488-labeled RNAP2 Ser2ph-Fab into living embryos (Fig. 1C, Movie 2). In good agreement with previous reports (Chan et al., 2019; Hadzhiev et al., 2019), RNAP2 Ser2ph foci were closely associated with bright miR-430 morpholino signals in living embryos (Fig. 1C, insets). Fab signals were less distinct than morpholino signals; this is likely due to weaker and more transient binding and/or binding to RNAP2 transcribing other genes. Morpholinos are known to bind target RNA with high affinity, leading to inhibition of translation (Summerton, 1999). In contrast, Fabs bind more transiently (Hayashi-Takanaka et al., 2011; Stasevich et al., 2014a) and appear not to inhibit transcription given the strong morpholino signal. Thus, the intranuclear distribution of RNAP2 Ser2ph-Fab can serve as an indicator of local transcription activity in living zebrafish embryos.

**Fig. 2.**
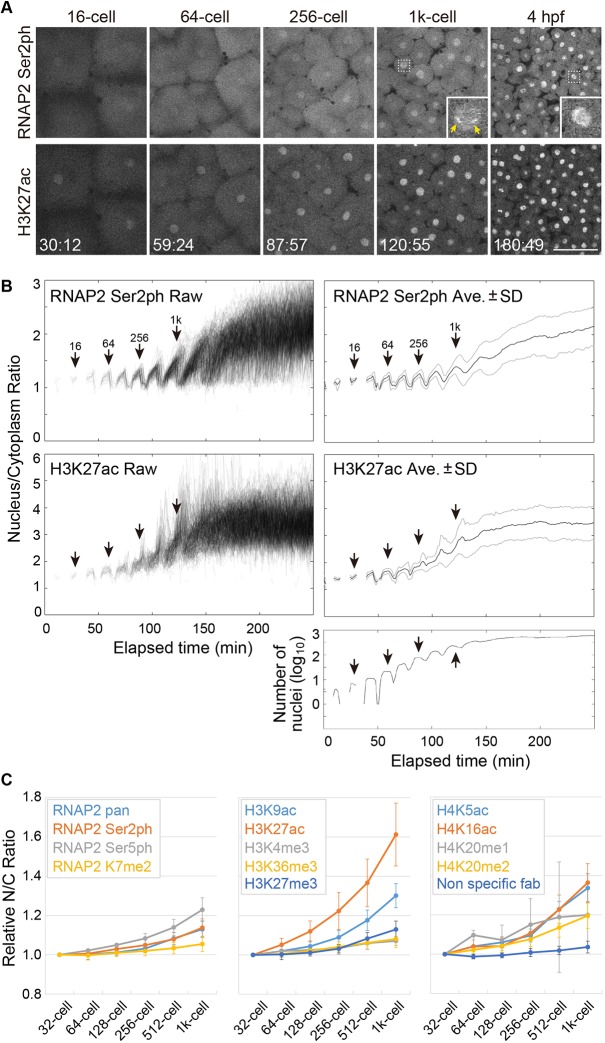
**Changes in the nuclear enrichments of Fabs specific to RNAP2 Ser2ph and H3K27ac during zebrafish embryo development.** (A) Representative images of embryos injected with Fabs specific for RNAP2 Ser2ph (Alexa 488), H3K27ac (Cy3) and H3K9ac (Cy5). Single confocal sections for RNAP2 Ser2ph and H3K27ac are shown. Insets show magnified views of the indicated areas. Yellow arrows indicate RNAP2 Ser2ph foci in nuclei. Elapsed times (min:s) are indicated. See also Movie 3. Scale bar: 100 μm. (B) Nucleus/cytoplasm intensity ratios. Stages judged from time-lapse images are indicated. A graph representing the number of measured nuclei is shown at the bottom. The global level of H3K27ac in nuclei increased slightly earlier than that of RNAP2 Ser2ph. (C) Changes in the nuclear enrichments of Fabs specific for various RNAP2 and histone modifications. Relative nucleus/cytoplasm (N/C) intensity ratios, relative to those of the 32-cell stage, are shown for various modifications and a control (mean±s.d. of 3 embryos).

**Fig. 3.**
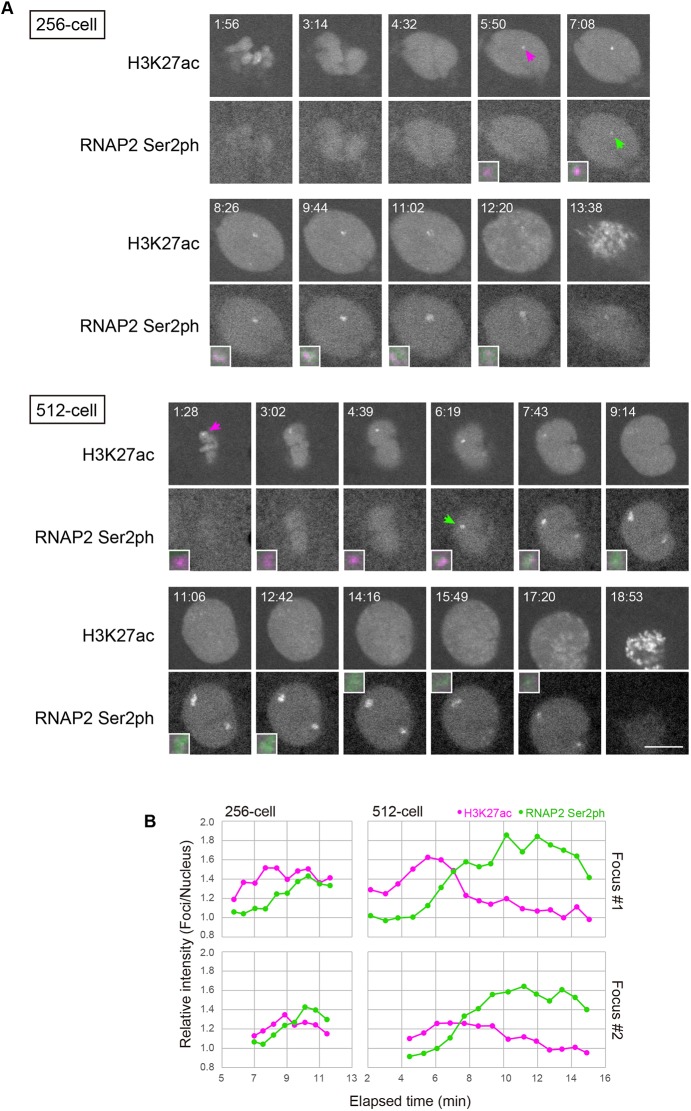
**Dynamics of histone H3K27ac and transcription foci at the 256- and 512-cell stages.** (A) Simultaneous visualization of RNAP2 Ser2ph and H3K27ac at the 256- and 512-cell stages. Embryos were injected with Fabs specific for RNAP2 Ser2ph (Alexa 488), H3K27ac (Cy3) and H3K9ac (Cy5). Single confocal sections for RNAP2 Ser2ph and H3K27ac are shown. RNAP2 Ser2ph accumulated close to H3K27ac foci. Arrows indicate H3K27ac and RNAP Ser2ph foci in the nucleus. Magnified and merged images of foci are shown in insets (H3K27ac, magenta; RNAP2 Ser2ph, green). Elapsed time (min:s) is indicated. See also Movies 6 and 7 for the 256- and 512-cell stages, respectively. Scale bar: 10 μm. (B) Relative intensity of foci. The intensity of H3K27ac and RNAP2 Ser2ph foci was measured and normalized to that of the whole nucleus to yield the foci/nucleus ratio.

The images acquired with light-sheet microscopy often suffer from striping artifacts (Rohrbach, 2009). To quantify how histone modifications change during ZGA, we used a confocal microscope, which yielded a higher spatial resolution and more homogenous background, albeit with a restricted field of view and a slower scanning speed. Time-lapse analysis showed that RNAP2 Ser2ph-Fabs became gradually concentrated in nuclei during embryo development (Fig. 2A, Movie 3), as observed using the light-sheet microscope. To quantify the changes in modification levels, we measured the N/C ratio (Hayashi-Takanaka et al., 2011; Stasevich et al., 2014b). The intensity on mitotic chromosomes was often difficult to measure because of their irregular shape and weak Fab enrichments, resulting in poor image segmentation during mitosis (Fig. 2B, Fig. S2). We therefore focused on Fab enrichments in interphase nuclei. Note that there was little enrichment of RNAP2 Ser2ph Fab on mitotic chromosomes (Movie 3; see also Fig. 3). The N/C ratio of RNAP2 Ser2ph was slightly increased at the 256-cell stage, and then became higher at the 1k-cell stage (Fig. 2B, Fig. S3), consistent with the timing of the major ZGA (Tadros and Lipshitz, 2009). Again, two bright foci were observed (Fig. 2A, inset at 1k-cell stage). Thus, quantifiable changes in the nuclear enrichment of RNAP2 Ser2ph-Fabs appear to reflect changes in the levels of transcription in zebrafish embryos to some extent.

### Quantifying histone modification changes

We next systematically analyzed the dynamics of relevant histone modifications. Among the various histone modifications investigated, H3K27ac exhibited the most drastic changes in early developmental stages. Its nuclear intensity continuously increased from the 64-cell stage, earlier than RNAP2 Ser2ph (Fig. 2B, Fig. S3). Modifications at other residues, such as H3K9ac and H4 Lys16 acetylation (H4K16ac), and RNAP2 Ser5ph, which is associated with transcription initiation (Zaborowska et al., 2016), also increased during embryo development, but not as highly as H3K27ac during the 64- to 128-cell stages (Fig. 2C; Fig. S4). Subtle increases after the 512-cell stage were also observed for methylation at various sites, including H3K4me3, a mark on the transcription start site of actively transcribed genes (Soares et al., 2017), H3 Lys27 trimethylation (H3K27me3), a mark associated with facultative heterochromatin (van Mierlo et al., 2019), and H3 Lys36 trimethylation (H3K36me3), a mark added with elongating RNAP2 (Li et al., 2019a) (Fig. 2C, Fig. S4). These data are largely consistent with immunofluorescence data using fixed cells (Zhang et al., 2018). The gradual increases in histone methylation are probably associated with the establishment of epigenomic states, depending on cell lineage and differentiation (Lindeman et al., 2011), an exception being H4K20me1, which showed drastic fluctuations throughout a single cell cycle (Fig. S4), reminiscent of its behavior in mammalian somatic cells (Rice et al., 2002; Sato et al., 2016). Finally, we confirmed our observations for RNAP2 Ser2ph and H3K27ac by immunofluorescence with fixed embryos at different stages using two independent antibodies (Figs S5 and S6). Taken together, these data suggest that the dynamic increase of H3K27ac might play a role in transcription activation during the minor and major ZGA, whereas H3K4me3 is more likely to be a pre-existing mark on target genes (Lindeman et al., 2010, 2011; Zhang et al., 2018).

### Histone H3K27ac precedes transcription

To clarify the relationship between H3K27ac and RNAP2 Ser2ph, we analyzed the dynamics of H3K27ac during the formation of RNAP2 Ser2ph foci (see Fig. 2A, inset of the 1k-cell image). The Fab probes for H3K27ac and RNAP2 Ser2ph have been well characterized and documented in living mammalian cells (Hayashi-Takanaka et al., 2011; Stasevich et al., 2014a). To follow the dynamics of those modifications, we acquired images at a higher magnification with shorter time intervals by scanning only a small area just covering a single nucleus from the 64-cell stage. In the 64- and 128-cell stages, H3K27ac foci appeared in nuclei during interphase, but concentration of RNAP2 Ser2ph was hardly observed (Fig. S7; Movies 4 and 5). In contrast, by the 256- and 512-cell stages, RNAP2 Ser2ph accumulated near H3K27ac foci, and became more concentrated within growing foci, whereas H3K27ac began to fade, probably as a result of chromatin unfolding and/or deacetylation (Fig. 3, Movies 6 and 7). The close association between RNAP2 Ser2ph and H3K27ac was confirmed by immunofluorescence using embryos that were injected with RNAP2 Ser2ph-Fab, fixed at the 512-cell stage, and stained with anti-H3K27ac antibody. In fixed embryos, H3K27ac signals were observed next to RNAP2 Ser2ph foci (Fig. S8). These data suggest that H3K27ac precedes RNAP2 transcription in miR-430 loci.

To rule out the possibility that our results were due to the relative affinity of Fab (Hayashi-Takanaka et al., 2011) and/or the sensitivity of the fluorescent dyes we used (Hayashi-Takanaka et al., 2014), we repeated experiments with differently labeled Fabs. To lower the affinity of H3K27ac-Fab, we increased the dye conjugation ratio, resulting in shorter binding times as revealed by a photobleaching assay, and swapped the fluorescence dyes between the two Fabs (Fig. S9). When focus formation was investigated at the 1k-cell stage using the various Fabs, enrichment of H3K27ac at the focus consistently peaked a few minutes prior to the RNAP2 Ser2ph peak (Fig. S10). It was, however, still possible that RNAP2 Ser2ph foci might be too faint to see at earlier time points, even though its accumulation peak was observed later than H3K27ac. In fact, immunofluorescence and RNA fluorescence *in situ* hybridization data indicate RNAP2 Ser2 foci and miR-430 transcripts as early as the 64-cell stage (Chan et al., 2019; Hadzhiev et al., 2019; Figs S5 and S6).

**Fig. 4.**
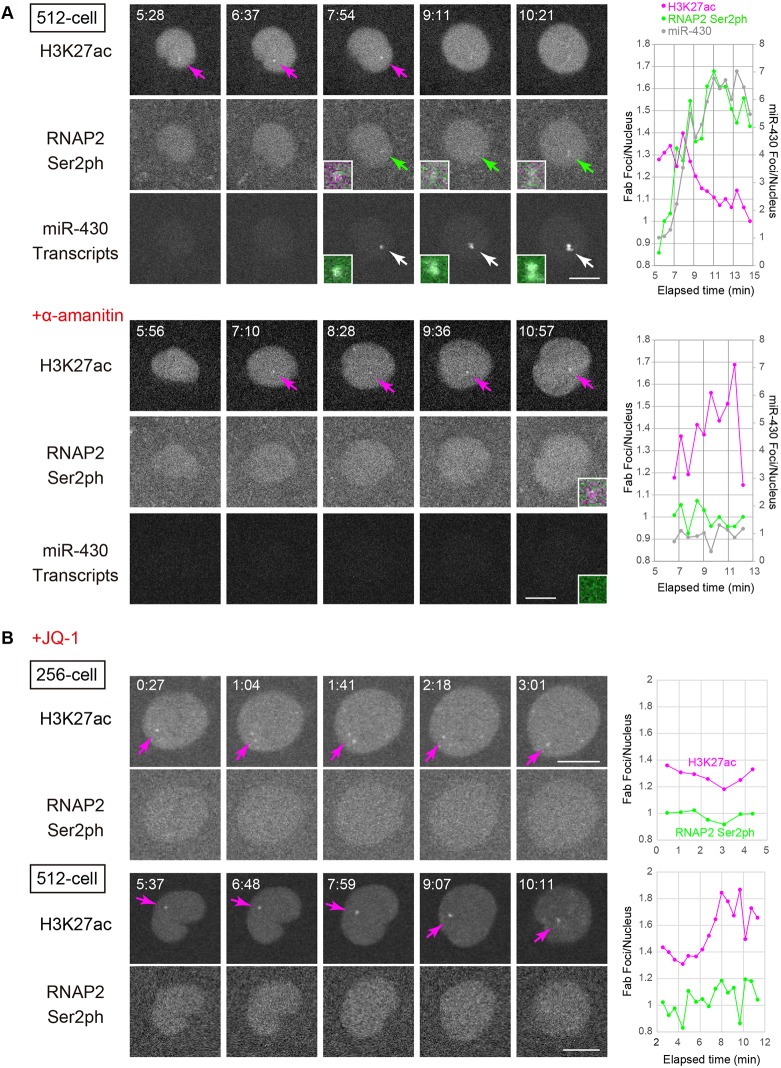
**α-Amanitin does not affect H3K27ac but JQ-1 inhibits RNAP2 Ser2ph foci.** (A) Effects of α-amanitin, an RNA polymerase inhibitor, on RNAP2 Ser2ph, H3K27ac, and miR-430 transcription. Embryos were injected with Fabs specific for RNAP2 Ser2ph (Alexa 488) and H3K27ac (Cy3) and miR-430 morpholino (Cy5). In some cases, the embryos were then injected with α-amanitin. Single confocal sections are shown. Arrows indicate H3K27ac, RNAP2 Ser2ph, and miR-430 transcript foci in nuclei. Magnified and merged images of foci are shown in insets (H3K27ac, magenta; RNAP2 Ser2ph, green; miR-430 morpholino, gray). Graphs on the right show the changes in relative focus intensities by time. The intensities of H3K27ac, RNAP2 Ser2ph, and miR-430 foci were measured and normalized to those of the whole nucleus to yield foci/nucleus ratios. After α-amanitin injection, RNAP2 Ser2ph and miR-430 morpholino were not accumulated in foci at the 512-cell stage, whereas H3K27ac still accumulated in foci. See also Movies 8 and 9 for embryos without and with α-amanitin, respectively. (B) Effects of JQ-1, a BET domain binder, on RNAP2 Ser2ph and H3K27ac. Embryos were injected with Fabs specific for RNAP2 Ser2ph (Alexa 488), H3K27ac (Cy3) and H3K9ac (Cy5), and then soaked in 10 μM JQ-1. Single confocal sections for RNAP2 Ser2ph and H3K27ac are shown. Graphs on the right show the changes in relative focus intensities by time, as in A. RNAP2 Ser2ph focus formation was inhibited by JQ-1. Elapsed time (min:s) is indicated. See also Movies 10 and 11 for the 256- and 512-cell stages, respectively. Scale bars: 10 μm.

We therefore attempted to perturb the transcription foci using chemical compounds to investigate the relationship between H3K27ac and transcription. We first used α-amanitin to inhibit RNAP2 transcription (Kane et al., 1996). As a result of α-amanitin treatment, miR-430 transcripts were no longer detected in the form of foci at 4 hpf (∼2k-cell stage), indicating that transcription was indeed abolished (Fig. S11). Also, the N/C ratio of RNAP2 Ser2ph Fab, but not that of H3K27ac, was decreased (Fig. S11). We then visualized RNAP2 Ser2ph and H3K27ac together with miR-430 morpholino without or with α-amanitin (Fig. 4A; Movies 8 and 9). In the control embryo without α-amanitin, RNAP2 Ser2ph and miR-430 morpholino became concentrated close to H3K27ac foci at the 512-cell stage. In the presence of α-amanitin, H3K27ac foci still appeared but RNAP2 Ser2ph and miR-430 morpholino were not concentrated in foci. These data indicate that H3K27ac levels can be increased without active RNAP2 transcription.

To investigate further the effect of H3K27ac on transcription, we initially attempted to inhibit p300 histone acetyltransferase using its selective inhibitor C646 (Bowers et al., 2010). Against our expectations, both the soaking of embryos in 10 μM C646 and the injection of 50 mM C646 into embryos did not affect H3K27ac levels at the foci. We next tried to inject multiple morpholinos specific to the acetyltransferases p300 and CBP, as described by Zhang et al. (2018). Again, though, we could not abolish H3K27ac at the foci when the morpholinos were injected into 1-cell-stage embryos sequentially with Fabs. This discrepancy might be due to the different timing of injection; Zhang et al. (2018) injected morpholinos into oocytes. Finally, we tried to use a bromodomain and extra-terminal motif (BET) inhibitor, JQ-1. JQ-1 treatment deters BET family proteins from binding to acetylated histones and may inhibit transcription activation (Lovén et al., 2013). To validate JQ-1 inhibition of the binding of BET family proteins in zebrafish, we injected mRNA encoding a fusion construct of bromodomain 2 from human BRD4 tagged with super-folder GFP and the nuclear localizing signal (BD2-sfGFP-NLS). The construct successfully detected the effect of JQ-1 in human cells (Fig. S12). In zebrafish embryos, BD2-sfGFP-NLS showed a subtle concentration on H3K27 foci, but 10 μM JQ-1 abolished such a concentration, suggesting that JQ-1 functions in zebrafish embryos (Fig. S13). Based on a decrease in miR-430 morpholino accumulation, transcription of miR-430 was also partially inhibited (Figs S13 and S14). Moreover, a higher concentration of JQ-1 almost abolished miR-430 transcription (Fig. S14). In the presence of 10 μM JQ-1, H3K27ac foci still appeared, but RNAP2 Ser2ph were scarcely concentrated in foci at the 256- and 512-cell stages (Fig. 4B, Movies 10 and 11). These results support the notion that acetyl-binding BET family proteins stimulate transcription activation in the miR-430 loci, consistent with recent work from other groups (Zhang et al., 2018; Chan et al., 2019).

## Conclusions

In this study, we used Fab-based imaging to monitor the dynamics of histone modifications during ZGA in living zebrafish embryos. This revealed spatiotemporal coordination within single nuclei, where H3K27ac was concentrated at distinct foci on the miR-430 gene cluster prior to its transcription. These observations, together with inhibitor assays, suggest that H3K27ac precedes active transcription during ZGA, probably mediated through acetyl-reader proteins, in good agreement with recent work on zebrafish (Zhang et al., 2018; Chan et al., 2019) and other systems (Stasevich et al., 2014a; Cho et al., 2018; Raisner et al., 2018). These observations illustrate the potential of our method to track the dynamics of RNAP2 and histone modifications during the development of living organisms. Further investigation of H3K27ac and RNAP2 Ser2ph during later stages will be interesting to provide a link between chromatin modifications, transcription, and nuclear organization (Hilbert et al., 2018preprint; Kaaij et al., 2018; Laue et al., 2019). As RNAP2 Ser2ph distribution changes from the accumulation at two miR-430 foci to more scattered patterns after the major ZGA, acquisition of higher spatial resolution and/or higher signal-to-noise images will be needed to follow the dynamic changes of individual gene loci. Although such high-resolution imaging is still challenging, this may be achievable using the latest microscopy techniques (Cho et al., 2018; Mir et al., 2018; Reisser et al., 2018; Li et al., 2019b). We therefore anticipate that the method presented here, which does not involve genetically recombinant materials, could be applied to a variety of developmental processes in any model and non-model organisms in the near future.

## MATERIALS AND METHODS

### Fabs

Fabs were prepared as previously described (Kimura and Yamagata, 2015) using mouse monoclonal antibodies specific to RNAP2 Ser2ph (CMA602/Pc26B5; IgG1) (Stasevich et al., 2014a,b), Ser5ph (CMA605/Pa57B7; IgG2b) (Stasevich et al., 2014a,b), K7me2 (CMA612/19B4; IgG1) (Dias et al., 2015), pan CTD (CMA601/C13B9; IgG1) (Stasevich et al., 2014a,b), H3K4me3 (CMA304/16H10; IgG1) (Kimura et al., 2008), H3K9ac (CMA310/19E5; IgG2a) (Hayashi-Takanaka et al., 2011), H3K27ac (CMA309/9E2H10; IgG1) (Kimura et al., 2008), H3K27me3 (CMA323/1E7; IgG1) (Hayashi-Takanaka et al., 2011), H3K36me3 (CMA333/13C9; IgG1) (Hayashi-Takanaka et al., 2011), H4K5ac (CMA405/4A7; IgG1) (Hayashi-Takanaka et al., 2015), H4K16ac (CMA416/1B2; IgG1) (Hayashi-Takanaka et al., 2015), H4K20me1 (CMA421/15F11; IgG1) (Hayashi-Takanaka et al., 2015) and H4K20me2 (CMA422/2E2; IgG1) (Hayashi-Takanaka et al., 2015). IgG was purified from hybridoma culture supernatant through a 1 ml Protein A-Sepharose column (GE Healthcare), as essentially described previously (Hayashi-Takanaka et al., 2011). Briefly, hybridomas were grown in CD Hybridoma medium (Invitrogen) supplemented with L-glutamine/penicillin/streptomycin (2 mM L-glutamine, 100 U/ml penicillin, 0.1 mg/ml streptomycin; Sigma-Aldrich). For purifying IgG1 subtype, the culture supernatant (250 ml) was filtrated through a 0.45-µm pore filter (Millipore Steritop Filter Unit), with added NaCl at a final concentration of 4 M and applied to a HiTrap Protein A HP Sepharose column (1 ml; GE Healthcare) using a peristaltic pump (ATTO; ∼1 ml/min flow rate). After washing the column with 20 ml Protein A IgG1 binding buffer (Thermo Fisher Scientific) using a syringe (∼1 ml/min flow rate), IgG was eluted using 5 ml Mouse IgG1 Mild Elution Buffer (Thermo Fisher Scientific) using a syringe (∼1 ml/min flow rate). After neutralizing the pH using 0.25 ml 1.5 M Tris-HCl (pH 8.8), the eluate was concentrated up to 4-8 mg/ml in PBS using an Amicon Ultra-15 filter unit 50k cut-off (Millipore) by centrifugation (5000 ***g***, 5×10-20 min). For purifying IgG2a and IgG2b subtypes, the filtered culture supernatant (250 ml) was directly applied to a HiTrap Protein A HP Sepharose column, and the column was washed with PBS.

Purified IgG was concentrated up to 4 mg in 0.5 ml digestion buffer (0.1 M sodium citrate, 5 mM EDTA, 25 mM cysteine, pH 6.0, for IgG1; 20 mM sodium phosphate, 10 mM EDTA, 20 mM cysteine for IgG2a and IgG2b) using an Amicon Ultra-15 filter unit 50k cut-off (Millipore; 5000 ***g***, 5×10-20 min), and digested with agarose beads conjugated with Ficin (Thermo Fisher Scientific; 1.2 mg Ficin/ml resin; 250 μl resin in a 500 μl reaction mixture for IgG1) or Papain (Thermo Fisher Scientific; 250 μg papain/ml; 250 μl resin in a 500 μl reaction mixture for IgG2a and IgG2b) for 4 h at 37°C. The mixture was transferred to an empty spin column (Harvard Apparatus; 731-1550) settled onto a 2 ml tube. After a brief spinning (5000 ***g***, 1 min), the filtrate was transferred to a new tube. To collect residual Fabs, beads were washed three times with 0.5 ml of 0.2 M sodium phosphate, 3 M NaCl (IgG1) or PBS (IgG2a and IgG2b). The bead-unbound fraction (total 2 ml) was applied to a 5 ml Protein A-Sepharose column (GE Healthcare) to remove Fc and undigested IgG using a syringe (∼1 ml/min flow rate). After leaving the column for 30 min at room temperature, unbound Fabs were collected by applying 15 ml of 0.2 M sodium phosphate, 3 M NaCl (IgG1) or PBS (IgG2a and IgG2b) by a syringe at an ∼1 ml/min flow rate. The resulting Fabs were concentrated up to ∼1 mg/ml in PBS using an Amicon Ultra-15 filter unit 10k cut-off (Millipore; 5000 ***g***, 5×10-20 min).

The concentration of Fab was measured using a spectrophotometer (NanoDrop) and a 100 μg aliquot was labeled with a sulfodichlorophenol ester of Alexa Fluor 488 (Thermo Fisher Scientific; 1-3 μl of 10 mg/ml DMSO solution), or N-hydroxysuccinimide ester of Cy3 (GE Healthcare; 1.5-3 μl of 10 mg/ml DMSO solution), Cy5 (GE Healthcare; 3-4 μl of 10 mg/ml DMSO solution), JF549 (Grimm et al., 2015; 1-2 μl of 10 mg/ml DMSO solution) and JF646 (Grimm et al., 2015; 1-2 μl of 10 mg/ml DMSO solution) in 0.1 M NaHCO_3_ (pH 8.3) in a 100 μl reaction mixture by incubating for 1 h at room temperature, as described previously (Kimura and Yamagata, 2015). The labeled Fabs were separated from free unreacted dyes using a gravity flow PD MiniTrap G-25 column pre-equilibrated with PBS (GE Healthcare), and were concentrated up to 1-2 mg/ml in PBS using an Amicon Ultra-0.5 unit 10k cut-off (Millipore; 12,000 ***g***, 2×5-10 min). Fab concentration and dye:protein ratio were measured using a spectrophotometer (NanoDrop) and Fabs labeled with <1.5 dye:protein ratio were used except in experiments for Figs S9 and S10. Normal mouse IgG (Jackson ImmunoResearch) was also processed using Ficin-agarose to yield a control Fab, which was then labeled with Alexa Fluor 488.

### Morpholino for detecting miR-430 transcripts

Morpholino antisense oligonucleotide to detect miR-430 was designed according to Hadzhiev et al. (2019) and 3′-primary amino-modified miR-430 morpholino (5′-TCTACCCCAACTTGATAGCACTTTC-3′) was obtained from Gene Tools LLC. For labeling with a fluorescent dye, 3 nmol of the morpholino was reacted with 50 μg of Cy3 or Cy5 NHS-ester (GE Healthcare; dissolved in methanol, aliquoted, and dried) in 10 μl of 0.1 M NaHCO_3_ (pH 8.3) for 1 h at room temperature. After the reaction, dye-conjugated morpholino was separated from free unreacted dyes using a gravity flow PD MiniTrap G-25 column (GE Healthcare) pre-equilibrated with PBS.

### Visualizing zebrafish embryogenesis

All zebrafish experiments were approved by the Tokyo Institute of Technology Genetic Experiment Safety Committee (I2018001) and animal handling was carried out according to the guidelines. We also complied with regulations of the animal ethics committees at the Howard Hughes Medical Institute Janelia Research Campus. Zebrafish (*Danio rerio*, AB) eggs were obtained from pairwise mating. Labeled Fabs (50-300 pg in ∼0.5 nl) and miR-430 morpholino (1.8 fmol in ∼0.5 nl) were injected into the yolk of 1-cell-stage embryos at room temperature (∼25°C). Embryos were kept at 28°C except during the period of manipulation, such as dechorionation and agarose embedding at ∼25°C, and during light-sheet imaging at 25°C (Fig. 1A) and 22°C (Fig. 1B).

For collecting images using simultaneous multi-view light-sheet microscopy (Tomer et al., 2012), injected embryos were dechorionated and embedded in 2-mm or 3-mm glass capillaries (Hilgenberg) filled with 0.9% low melting temperature agarose (Type VII, Sigma-Aldrich) prepared in zebrafish system water. The fully gelled agarose cylinder was extruded from the capillary to expose the embryo to the detection system. The capillary was then mounted vertically in the recording chamber containing zebrafish system water, so that the agarose section containing the embryo was mechanically supported by the glass capillary below with the animal pole of the embryo facing the microscope's detection arm. Bi-directional scanned laser light sheets at 488, 561 and 647 nm wavelengths were used for excitation. Fluorescence was detected using 525/50 nm band-pass, 561 nm long-pass, and 647 nm long-pass detection filters (Semrock), respectively. Imaging was performed using Nikon 16×/0.8 NA (Fig. 1B) or Zeiss 20×/1.0 NA (Fig. 1C) water-immersion objectives and images were acquired with Hamamatsu ORCA Flash 4.0 v2 sCMOS cameras. Time-lapse imaging was performed at a time interval of 1 min using the AutoPilot framework for automatic adaptive light-sheet adjustment. Image stacks of 198 planes encompassing the entire volume of the embryo with an axial step size of 2.031 µm were acquired for each time point. The lateral pixel sizes in the image data were 0.406 µm (Fig. 1B) or 0.325 µm (Fig. 1C).

For collecting images using a confocal microscope (FV1000; Olympus), injected embryos were incubated at 28°C until the 4-cell stage. The embryos were dechorionated and embedded in 0.5% agarose (Sigma-Aldrich, A0701) in 0.03% sea salt with the animal pole down on a 35-mm glass-bottom dish (MatTek), which was set on to a heated stage (Tokai Hit) at 28°C. Fluorescent images were acquired using an FV1000 (Olympus) operated by the built-in software FLUOVIEW ver.4.2 with a UPLSAPO 30× silicone oil immersion lens (NA 1.05), using 512×512 pixels, scan speed 2.0 μs pixel dwell time, zoom 1.0, and 4 μm *z*-interval (20-25 sections), with 488 nm (75 μW at the specimen), 543 nm (40 μW) and 633 nm (55 μW) laser lines.

For collecting live snapshot images in Figs S11 and S14, a spinning disk confocal system (Nikon Ti-E attached with Yokogawa CSU-W1, operated under NIS Elements ver. 5.11.01) equipped with a Plan Apo 20×(NA 0.74) lens, an electron multiplying charge-coupled device (EM-CCD; Andor Technology; iXon Life) and a laser unit (405- and 488-nm; Andor Technology; ILE, operated by Andor iQ3) was used.

### Quantification of RNAP2 Ser2ph and H3K27ac levels by immunofluorescence

For immunofluorescence in Figs S5 and S6, embryos were fixed at desired stages with 4% paraformaldehyde containing 0.1% Triton X-100 and 250 mM HEPES-HCl (pH 7.4) overnight at 4°C. After washing with PBS three times, embryos were dechorionated and deyolked manually using tweezers to obtain blastomeres. The blastomeres were permeabilized with 1% Triton X-100 for 1 h at room temperature and blocked with Blocking One P (Nacalai Tesque) for 1 h at room temperature. For staining with in-house antibodies (RNAP2 Ser2ph and H3K27ac; CMA602 and CMA309, respectively), blastomeres were incubated with 2 μg/ml Alexa Fluor 488-conjugated antibodies in 10% Blocking One P (Nacalai Tesque) in PBS containing 1 μg/ml Hoechst 33342 for 16 h at 4°C. For staining with commercial antibodies (RNAP2 Ser2ph and H3K27ac; ab5095 and ab177178, respectively; Abcam), blastomeres were incubated with 2 μg/ml antibodies in 10% Blocking One P in PBS for 40 h at 4°C. After washing with PBS three times, blastomeres were incubated with Alexa Fluor 488-conjugated anti-rabbit secondary antibody (Jackson ImmunoResearch; 711-005-152) in 10% Blocking One P in PBS containing 1 μg/ml Hoechst 33342 at 4°C for 16 h. After washing with PBS three times, blastomeres were embedded in 0.5% low-gelling temperature agarose (Sigma-Aldrich; A0701) with animal pole down on a 35-mm glass-bottom dish (IWAKI). Fluorescence images were acquired using a spinning disk confocal system described above; 30-46 *z*-stack images with 2 μm intervals were collected. The nuclear areas of individual cells were selected using Hoechst 33342 signals for thresholding and Alexa Fluor 488 intensities were measured using Fiji/ImageJ ver. 1.51f (http://fiji.sc/). Box plots were generated using BoxPlotR (http://shiny.chemgrid.org/boxplotr/). For statistical analysis, one-way analysis of variance (for three groups; Fig. S1) and unpaired two-tailed Student's *t*-test (for two groups; Fig. S11) were used (Microsoft Excel 2016).

### Fluorescence recovery after photobleaching

HeLa cells were routinely maintained in Dulbecco's modified Eagle's medium (DMEM; Nacalai Tesque) supplemented with L-glutamine/penicillin/streptomycin (2 mM L-glutamine, 100 U/ml penicillin, 0.1 mg/ml streptomycin; Sigma-Aldrich) and 10% fetal bovine serum (FBS; Thermo Fisher Scientific) at 37°C under 5% CO_2_ atmosphere with constant humidity. For Fig. S9, cells were plated on a 35-mm glass-bottom dish (IWAKI). The next day, 3 μl of 1-2 mg/ml Alexa Fluor 488- or Cy3-labeled Fab specific to H3K27ac was loaded into cells using glass beads (Sato et al., 2018). The medium was replaced with FluoroBrite (Thermo Fisher Scientific) containing L-glutamine/penicillin/streptomycin and FBS and cells were incubated for 3-4 h. Cells were then set onto a heating unit (Tokai Hit; 37°C) with a CO_2_-control system (Tokken) on a confocal microscope (Olympus FV1000) operated by built-in FV1000 software (ver. 4.2) with a 60× PlanApoN (NA 1.40) oil lens. For Alexa Fluor 488-labeled Fabs, 100 images were collected using a main scanner (0.4% 488-nm laser transmission; 2 μs/pixel; 256×256 pixels; pinhole 800 μm; 8× zoom; ∼468 ms/frame), and after collecting ten images, a ∼2 μm diameter spot was bleached using a second scanner (90% 405-nm laser transmission; 55 ms). For Cy3-labeled Fabs, images were collected using a main scanner (65% 543-nm laser transmission; 2 μs/pixel; 256×256 pixels; pinhole 800 μm; 8× zoom; ∼577 ms/frame) and the same main scanner (100% 488-nm laser transmission; ∼106 ms) was used for bleaching. The fluorescence intensities of the bleached area were measured using Fiji/ImageJ ver. 1.51f, and, after background subtraction, intensities relative to the averages before bleaching were obtained. Using the ‘Curve Fitting’ tool, the recovery curves (from four frames after bleaching) were fitted with the double exponential association kinetics *I*=*P_1_*×[1−exp(−*k_1_*×t)]+*P_2_*×[1−exp(−*k_2_*×t)]+*C*, where *I* is the relative intensity, *P_1_* the plateau value of population 1, *k_1_* the dissociation coefficient of population 1, *t* the time since photobleaching, *P_2_* the plateau value of population 2, *k_2_* the dissociation coefficient of population 2, and *C* the baseline (unbleached fraction) (Kimura and Cook, 2001). The half-time recovery was calculated using the following formula: *t_1/2_*=*P_1_*/(*P_1_*+*P_2_*)×ln(1/2)/*k_1_*+*P_2_*/(*P_1_*+*P_2_*)−ln(1/2)/*k_2_*.

### Inhibitors

α-Amanitin [∼0.25 ng in ∼0.5 nl water; Merck Millipore; a similar concentration (0.4 ng in 2 nl) was used by Kane et al., 1996] was injected into the embryo after injection of fluorescent probes. C646 (Calbiochem; 50 mM in DMSO) and JQ-1 (BPS Bioscience; 50 mM in DMSO) were added into embryo culture and embedding agarose at 10 μM.

### Construction and evaluation of sfGFP-tagged bromodomain (BD2-sfGFP-NLS)

To visualize bromodomain dynamics in zebrafish embryos, mRNA for super-folder GFP (sfGFP)-tagged bromodomain was prepared. A cDNA fragment encoding the human bromodomain BRD4 (GenBank Accession: NM_058243.2) was amplified from a HaloTag BRD4 expression vector (Promega; pFN21AE9668, 352-456 aa). Zebrafish nucleoplasmin 2b (GenBank Accession: NM_001123007) was cloned from zebrafish 24 hpf embryo cDNA and 41 amino acids of the C-terminal region (KKVTKNSAGKRKKPEKGEDEEASDGENPPKKGKGRGRKAKA) were used as a nuclear localization signal (NLS). To construct sfGFP-tagged bromodomain expression vector (pcDNA3-BD2-sfGFP-NLS-polyA), bromodomain, super-folder GFP (Addgene #54579, deposited by Michael Davidson and Geoffrey Waldo; Pédelacq et al., 2006), and NLS fragments were linked and inserted into a pcDNA3-based plasmid containing T7 promoter and poly A (Yamagata et al., 2005).

The acetyl-specific binding was evaluated using human osteosarcoma U2OS cells, which were routinely maintained in DMEM supplemented with L-glutamine/penicillin/streptomycin (2 mM L-glutamine, 100 U/ml penicillin, 0.1 mg/ml streptomycin; Sigma-Aldrich) and 10% fetal bovine serum (FBS; Thermo Fisher Scientific) at 37°C under 5% CO_2_ atmosphere with constant humidity. For live-cell imaging (shown in Fig. S12), cells were plated on a 35-mm glass-bottom dish (IWAKI). The next day, cells were transfected with pcDNA3-BD2-sfGFP-NLS-polyA using FuGene HD transfection reagent (Promega) according to the instruction manual (1 μg DNA and 3 μl FuGene HD were mixed in 100 μl Opti-MEM; Thermo Fisher Scientific). Twenty-four hours after transfection, the medium was replaced with FluoroBrite (Thermo Fisher Scientific) containing L-glutamine/penicillin/streptomycin, 10% FBS, 0.1 μM trichostatin A (TSA), and 0.1 μg/ml Hoechst 33342 for 4 h, before placing onto a heating unit (Tokai Hit; 37°C) with a CO_2_-control system (Tokken) on a confocal microscope (Olympus FV1000). Before and after addition of 10 μM JQ-1, fluorescence images were collected using built-in FV1000 software (ver. 4.2) equipped with a 60× PlanApoN (NA 1.40) oil lens (0.2% 405- and 1% 488-nm laser transmission; 4 μs/pixel; 800×800 pixels; pinhole 100 μm; 1.5× zoom; 2× averaging;∼21 s/frame).

### mRNA preparation and injection

pcDNA3-BD2-sfGFP-NLS-polyA was linearized with XhoI and treated with proteinase K. After purification of the linearized template by phenol/chloroform extraction and ethanol precipitation, *in vitro* transcription was performed using mMESSAGE mMACHINE (Thermo Fisher Scientific). RNA was purified by LiCl precipitation and re-suspended in H_2_O.

Before microinjection, zebrafish eggs were dechorionated manually using tweezers. A mixture (∼0.5 nl in PBS) of BD2-sfGFP-NLS mRNA (375 pg) and Cy5-labeled miR430-MO (0.8 pg) was injected into the yolk of 1-cell-stage embryos. Five minutes after mRNA injection, Cy3-labeled Fab specific to H3K27ac was injected (100 pg in ∼0.5 nl). Injected embryos were incubated at 28°C until the 4-cell stage and embedded in 0.5% low-gelling temperature agarose in 0.03% sea salt with animal pole down on a 35-mm glass-bottom dish. Fluorescence images were collected as described above.

### Quantification of fluorescence signals

To obtain time courses of N/C ratios, an existent pipeline for time-resolved, single nucleus level analysis was modified and extended (Joseph et al., 2017). In brief, the nuclear signal of the Cy5-labeled Fabs specific for histone H3 Lys9 acetylation (H3K9ac), which is a broad euchromatin mark, was used to segment and track individual nuclei during interphase. A manual review step on the basis of a graphical user interface was added to correct tracking errors. Based on single nuclei segmentation masks, the intra-nuclear and cytoplasmic intensities were quantified using two derivative segmentation masks. The cytoplasmic segmentation masks were generated by repeated dilation operations to a distance range of 8.3 µm around a given nucleus, with an inner zone of 3.3 µm around that was not included in the cytoplasmic masks. The intensity data of single cells were exported to tabulated files that can be accessed and further analyzed using other software tools, e.g. Microsoft Excel. All image analysis procedures were implemented in MatLab, using the BioFormats Open Microscopy Environment importer bfMatlab (Goldberg et al., 2005) and are available as open source code (https://github.com/lhilbert/NucCyto_Ratio_TimeLapse). As the size of raw data (several hundreds of gigabytes) makes permanent hosting impractical, raw data are available on request. For Fig. 3B, single *z*-planes in which the sectional area of H3K27ac focus became maximum were selected at each time point from time-lapse images. The regions of interest (ROIs) of 1 μm diameter circle just covering a H3K27ac focus on these planes were obtained using Fiji. All intensities in the ROI were summed.

## Supplementary Material

Supplementary information
