# Supplementary material for: Histone H3K27 acetylation precedes active transcription during zebrafish zygotic genome activation as revealed by live-cell analysis
Source: Development. 2019 Sep 30;146(19):dev179127. doi: 10.1242/dev.179127 (PMC6803375; doi:10.1242/dev.179127)
Supplement: Supplementary information [file develop-146-179127-s1.pdf]

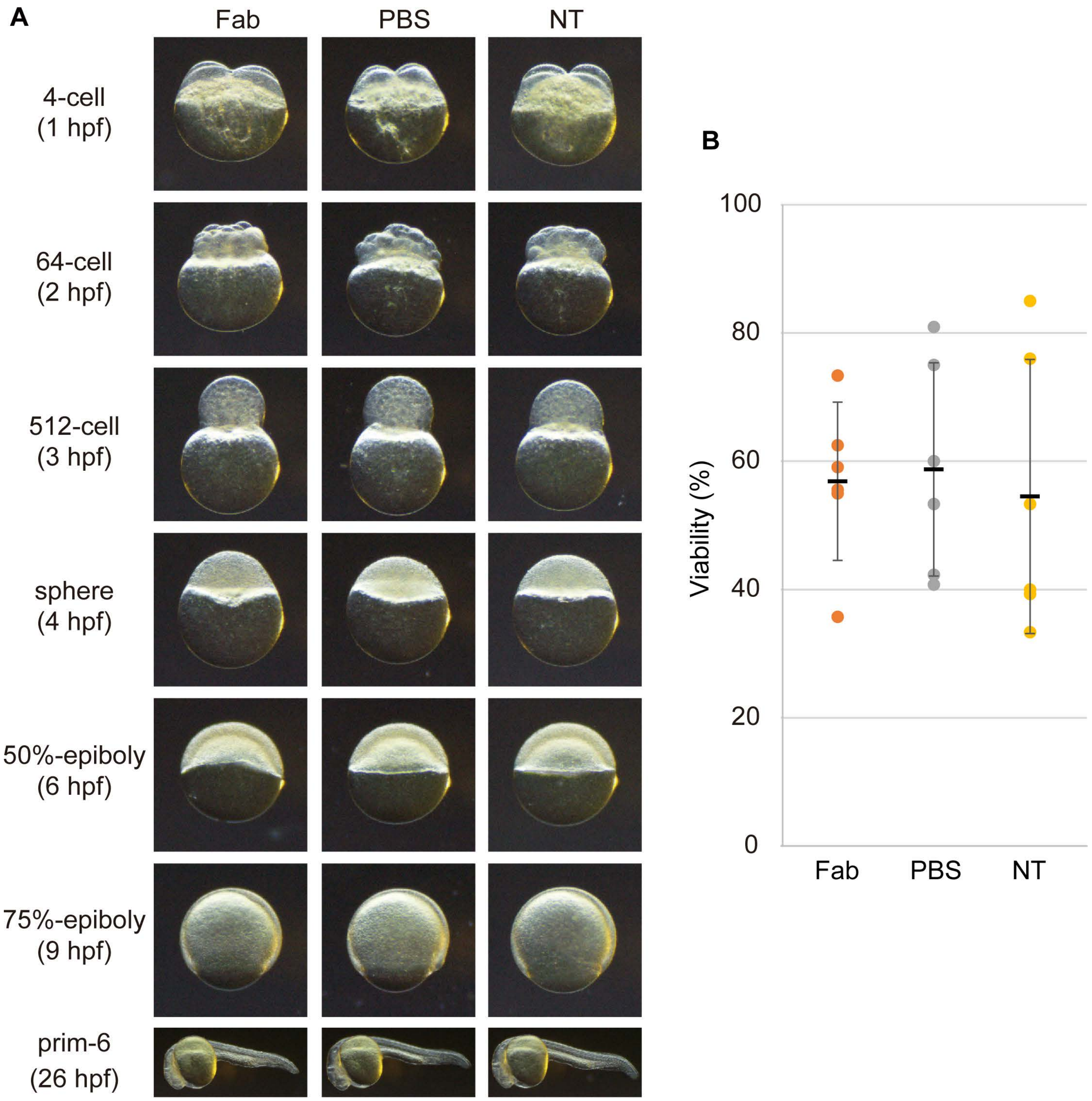

Figure S1. The influence of Fab injection on embryogenesis.
(A) Images of the embryos injected with Alexa488-labeled RNAP2 Ser2ph-Fab (Fab) or PBS, and non-treated (NT) embryos, are shown. (B) The percentages of viable embryos on the next day of injection from 6 independent experiments (15-30 embryos for each) are plotted. There was no significant difference ($p = 0.913$) among the samples by one-way analysis of variance.

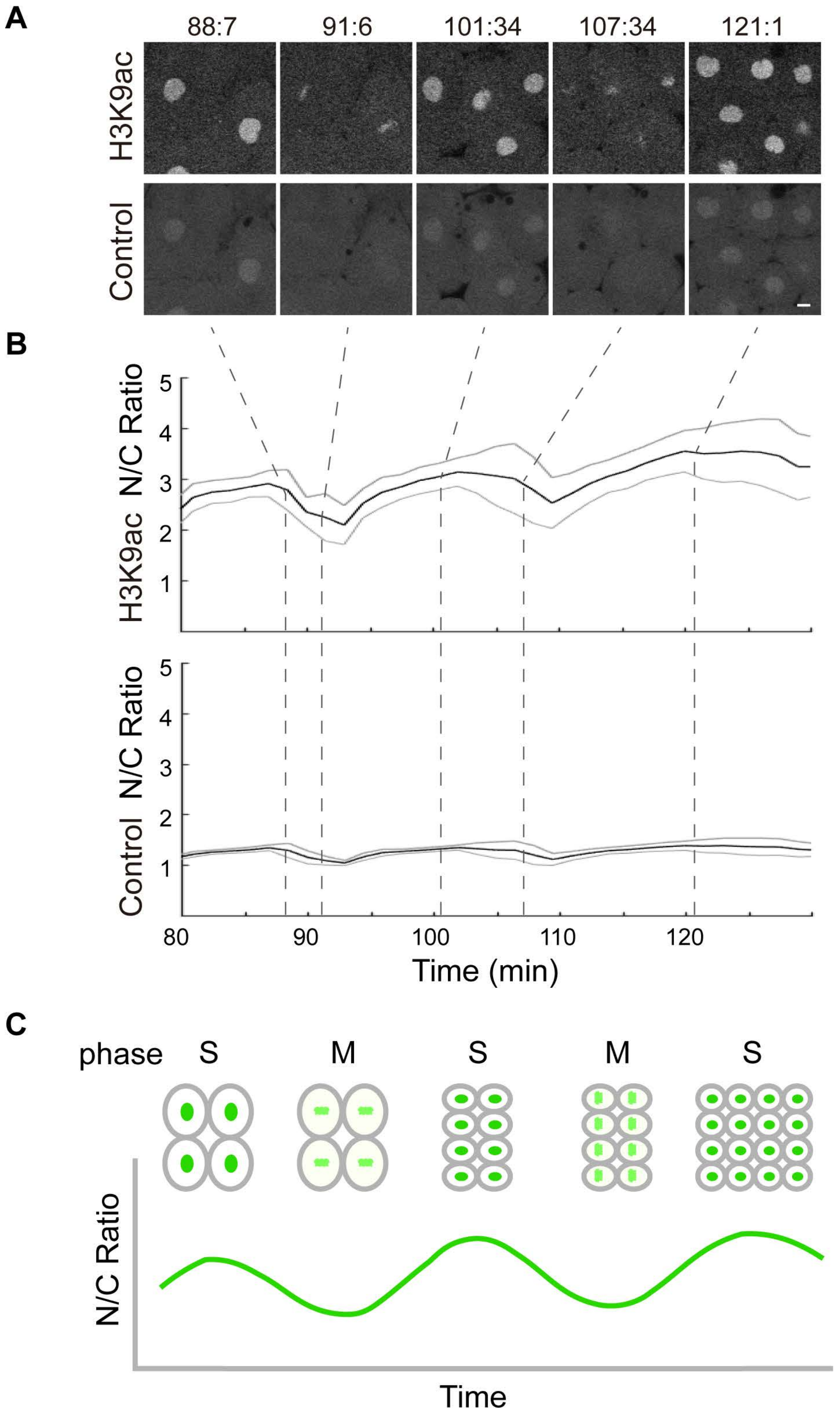

Development • Supplementary information

Figure S2. Oscillation of Fab concentration in the nucleus or condensed chromosomes during the cell cycle.

(A) The embryo injected with H3K9ac-Fab (Cy5) and non-specific Fab (Alexa488; Control) were imaged. Elapsed times (min:sec) are indicated. Scale bar, 10 μm. (B) Nucleus/Cytoplasm (N/C) intensity ratio of H3K9ac and Control Fab. Average ± Standard deviation is shown. (C) Schematic illustration for the oscillation of N/C ratio during the cell cycle.

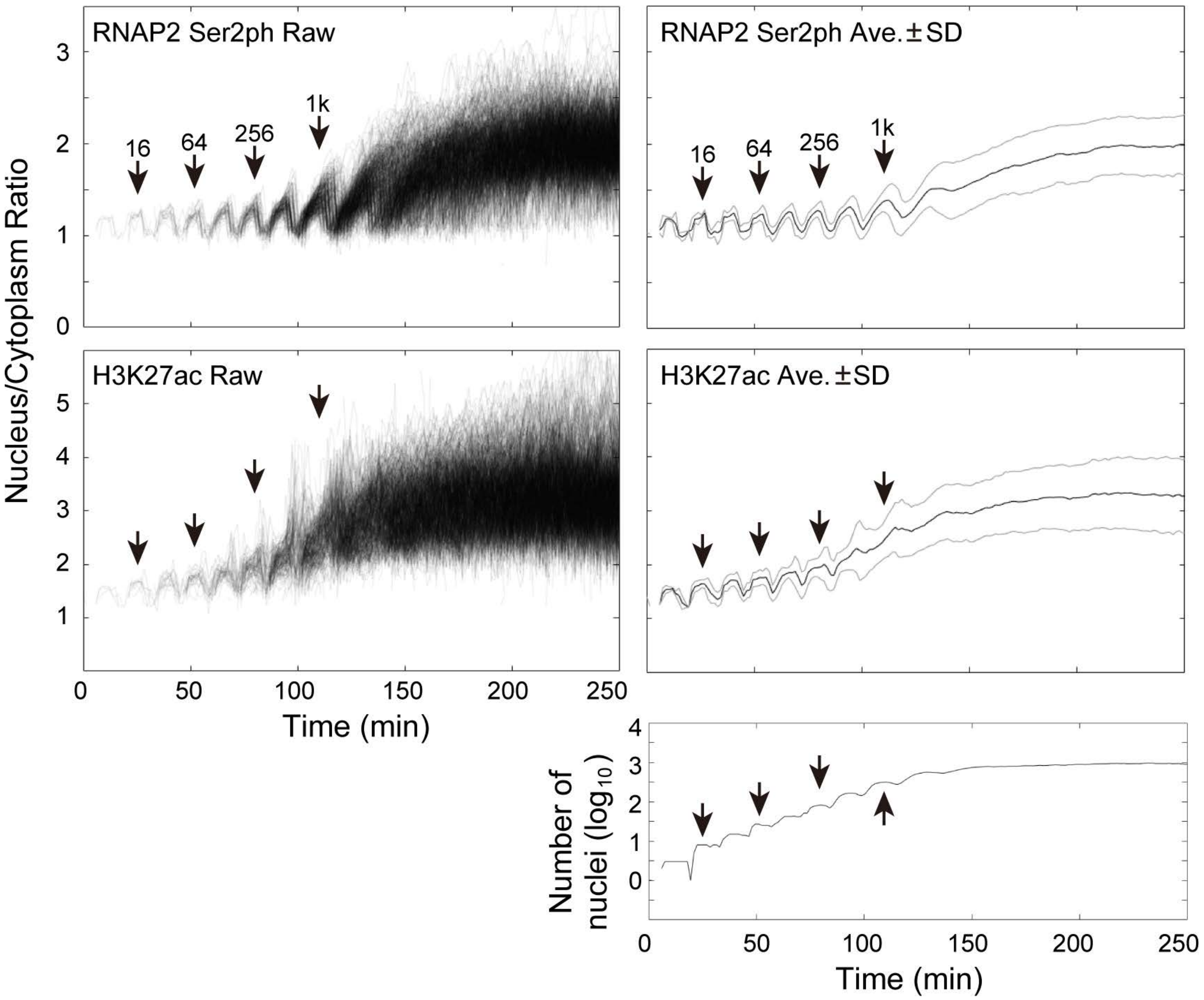

RNAP2 Ser2ph Raw
RNAP2 Ser2ph Ave.±SD
H3K27ac Raw
H3K27ac Ave.±SD
16
64
256
1k
Nucleus/Cytoplasm Ratio
Time (min)
Number of nuclei (log_{10})

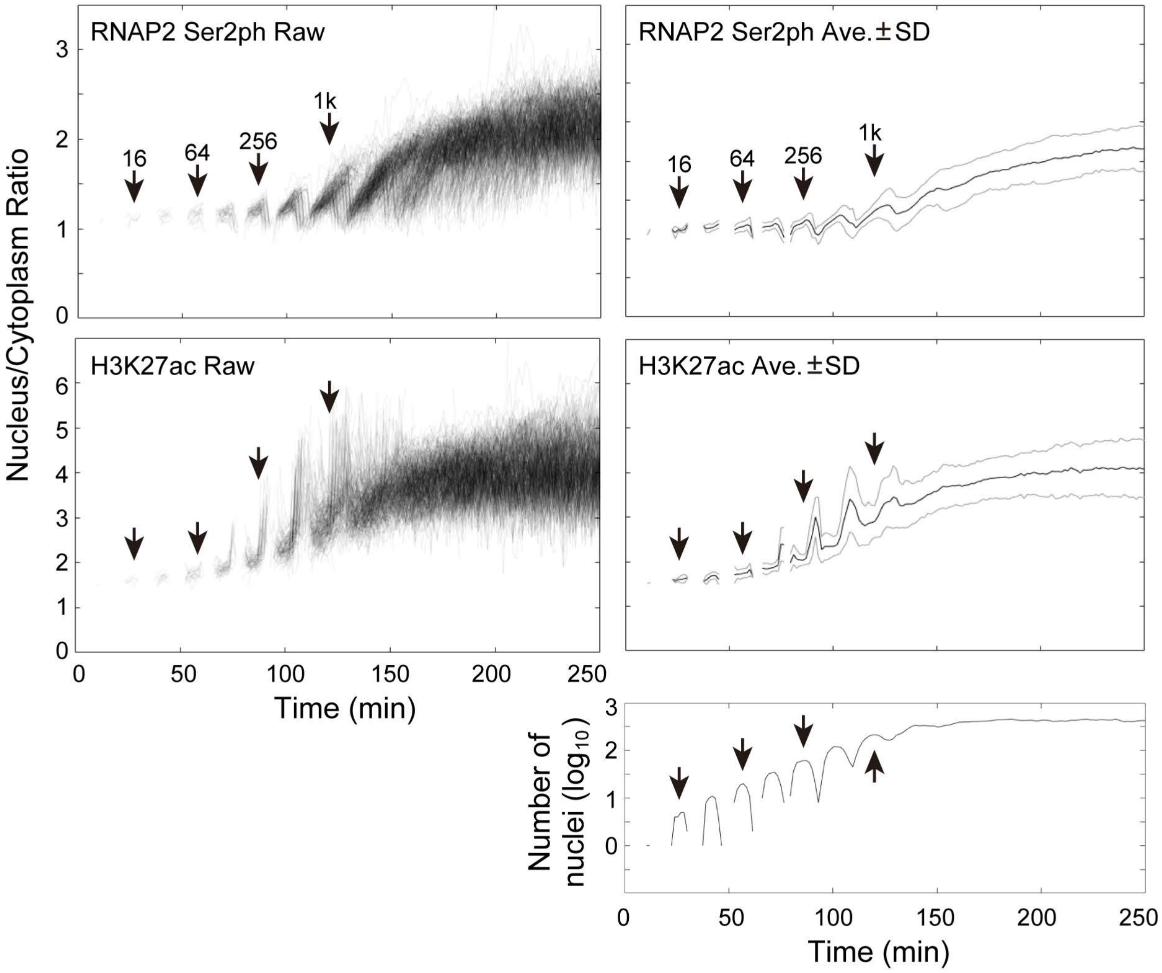
RNAP2 Ser2ph Raw
RNAP2 Ser2ph Ave.±SD
H3K27ac Raw
H3K27ac Ave.±SD
16
64
256
1k
Nucleus/Cytoplasm Ratio
Time (min)
Number of nuclei (log_{10})

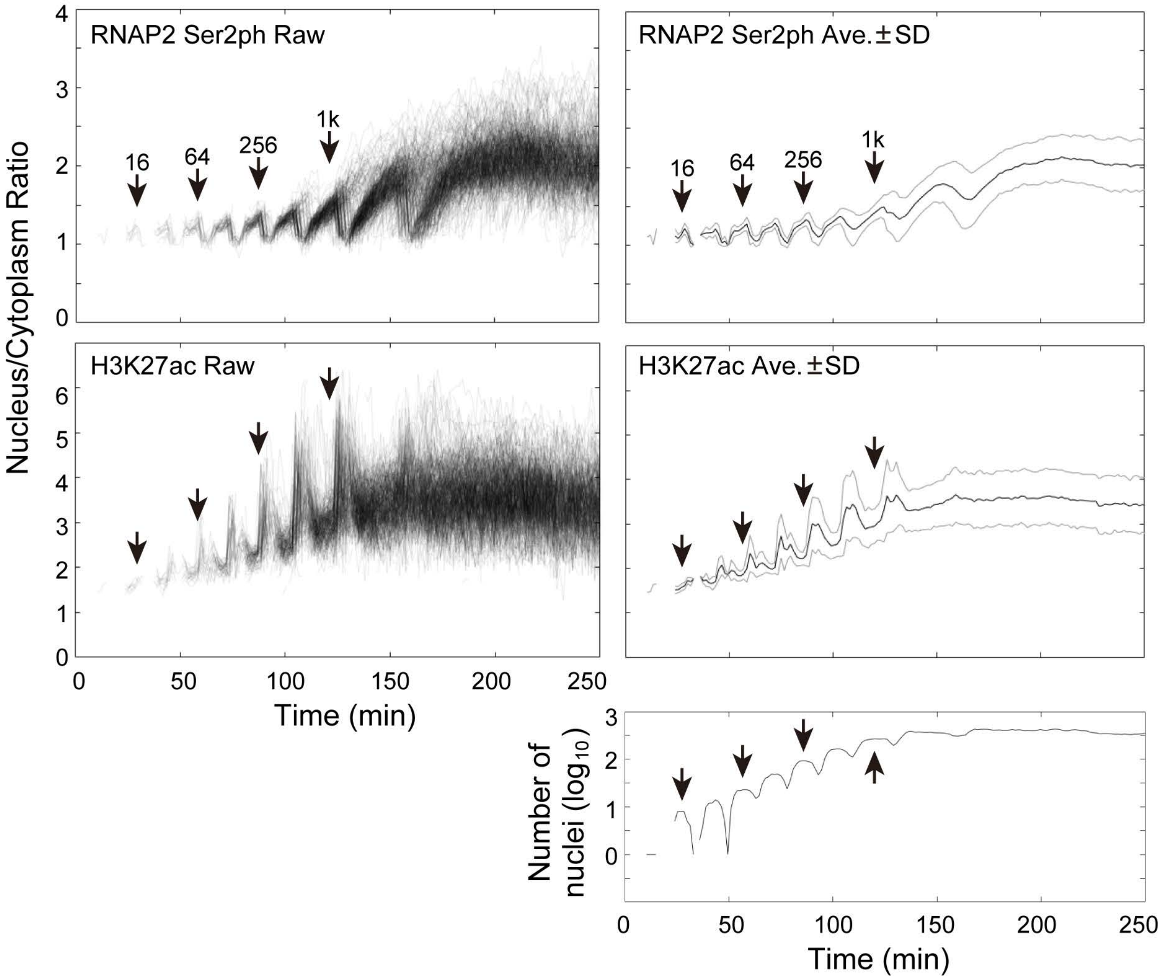
RNAP2 Ser2ph Raw
RNAP2 Ser2ph Ave.±SD
H3K27ac Raw
H3K27ac Ave.±SD
16
64
256
1k
Nucleus/Cytoplasm Ratio
Time (min)
Number of nuclei (log_{10})

Figure S3. Nucleus/Cytoplasm intensity ratios of Fabs specific to RNAP2 Ser2ph and H3K27ac in developing zebrafish embryos.

Embryos were injected with Fabs specific to RNAP2 Ser2ph (Alexa488), H3K27ac (Cy3), and H3K9ac (Cy5). N/C ratios of RNAP2 Ser2ph and H3K27ac (left, individual nuclei; right, averages with standard deviations) from there embryos are shown, with the number of measured nuclei (bottom).

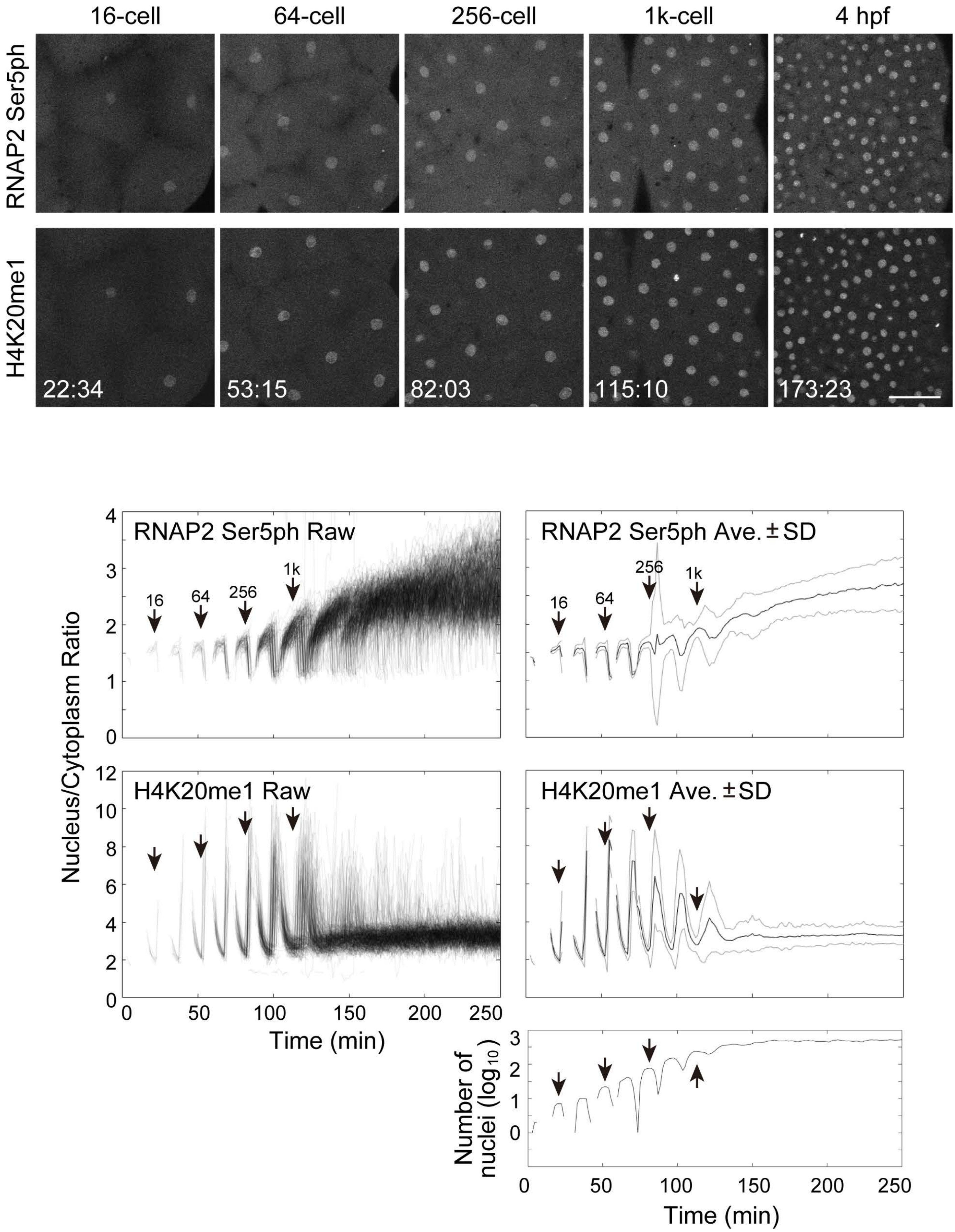

Development • Supplementary information

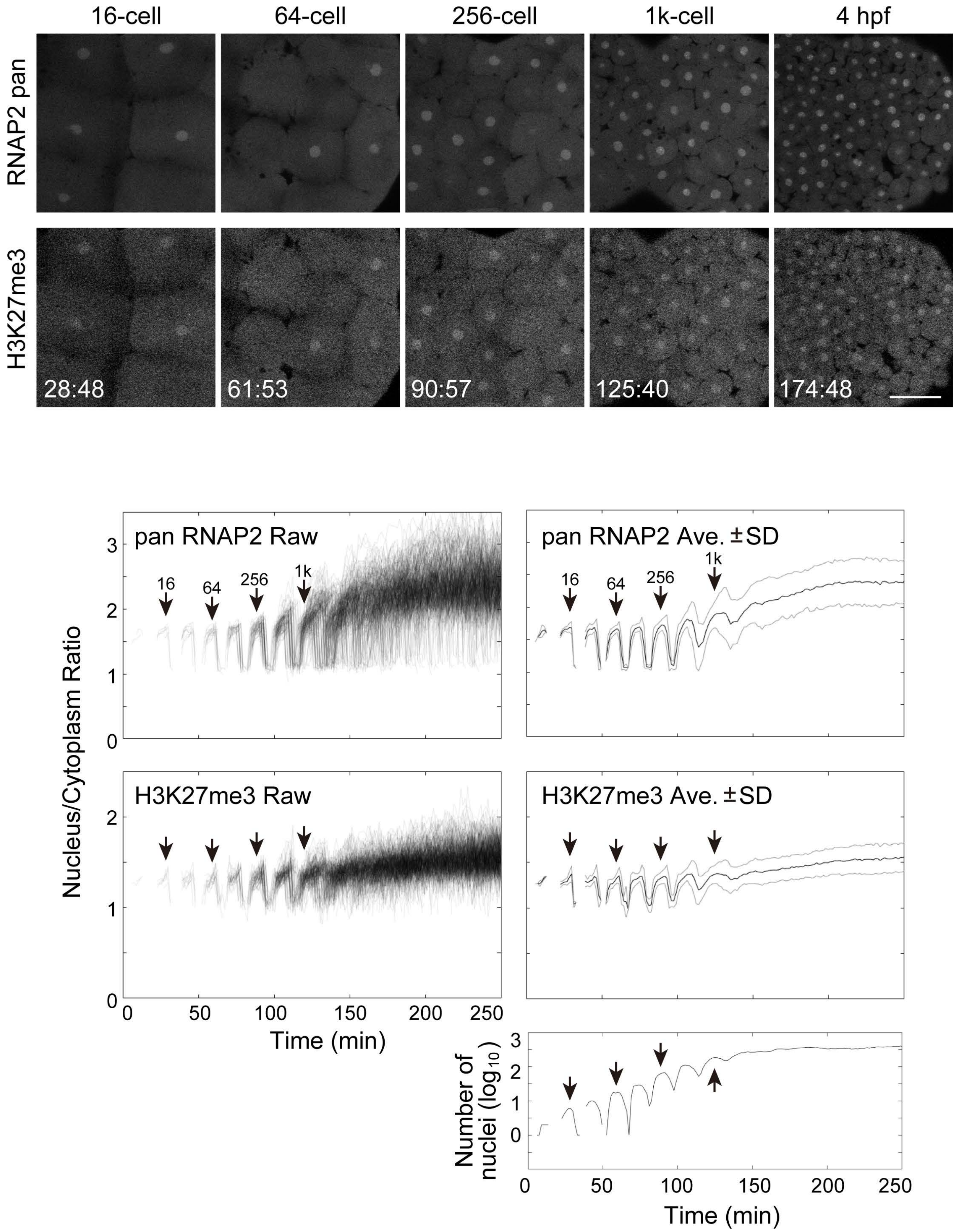

Development • Supplementary information

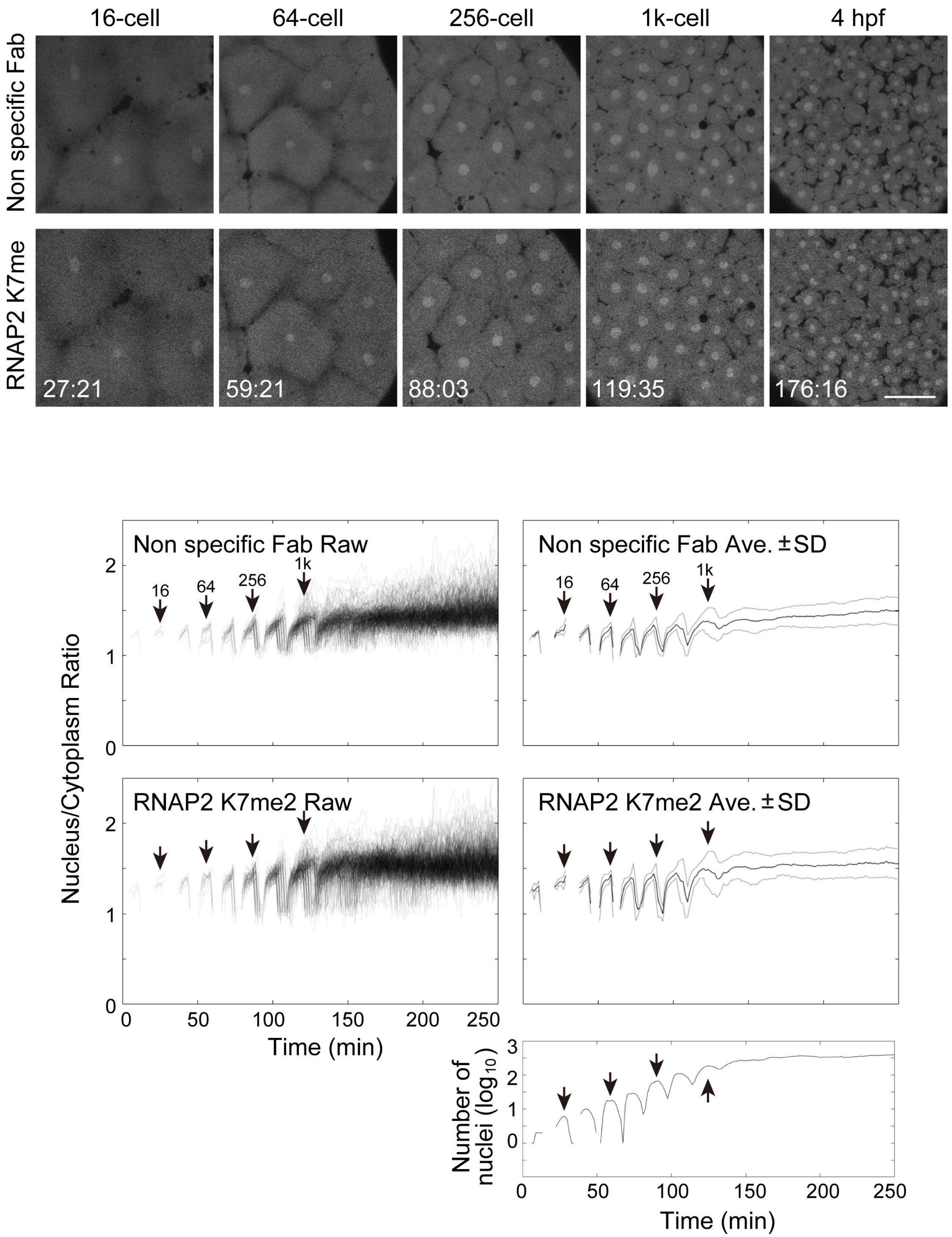

Development • Supplementary information

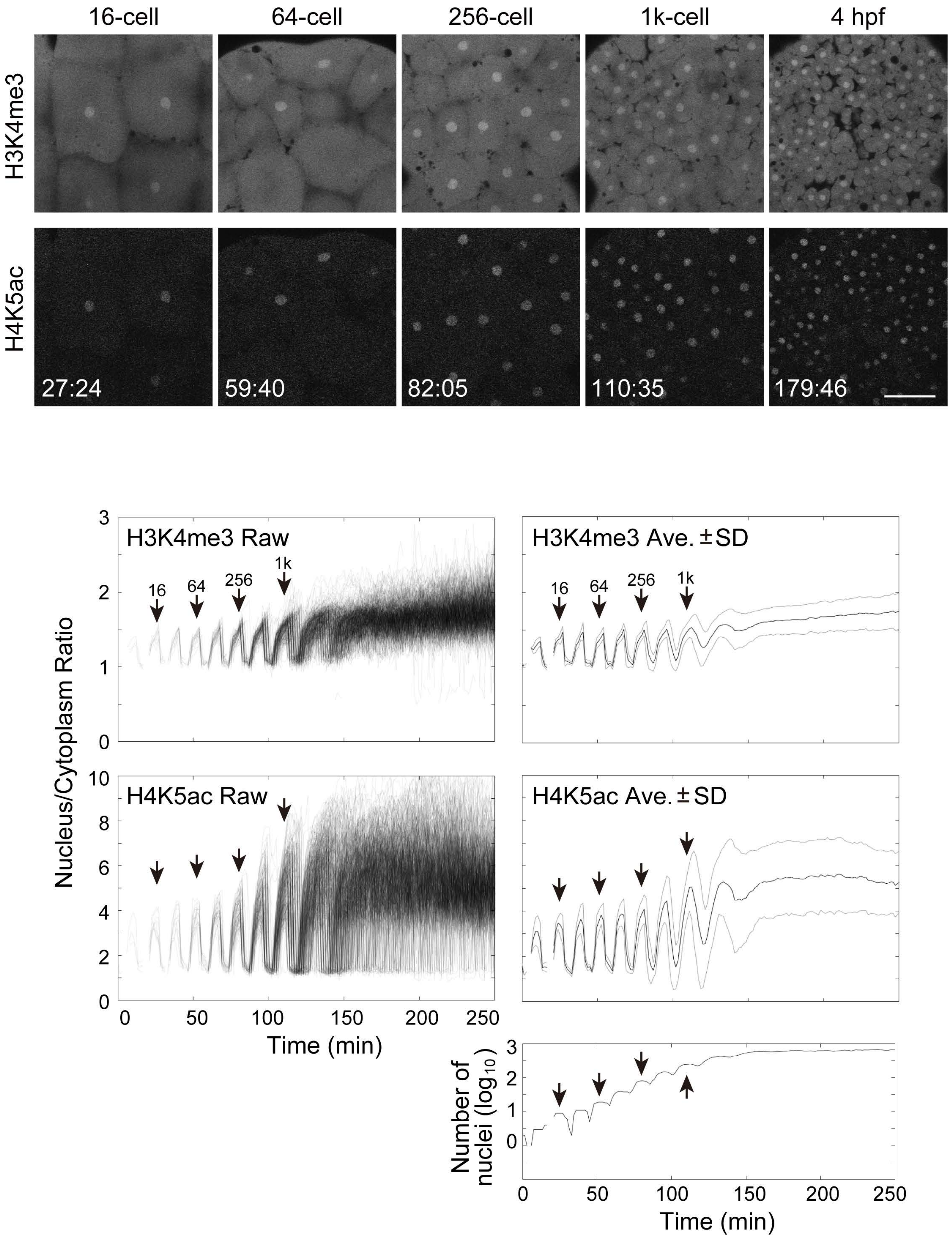
16-cell
64-cell
256-cell
1k-cell
4 hpf
H3K4me3
H4K5ac
27:24
59:40
82:05
110:35
179:46
H3K4me3 Raw
16
64
256
1k
H3K4me3 Ave. ± SD
H4K5ac Raw
H4K5ac Ave. ± SD
Nucleus/Cytoplasm Ratio
Time (min)
Number of nuclei (log10)

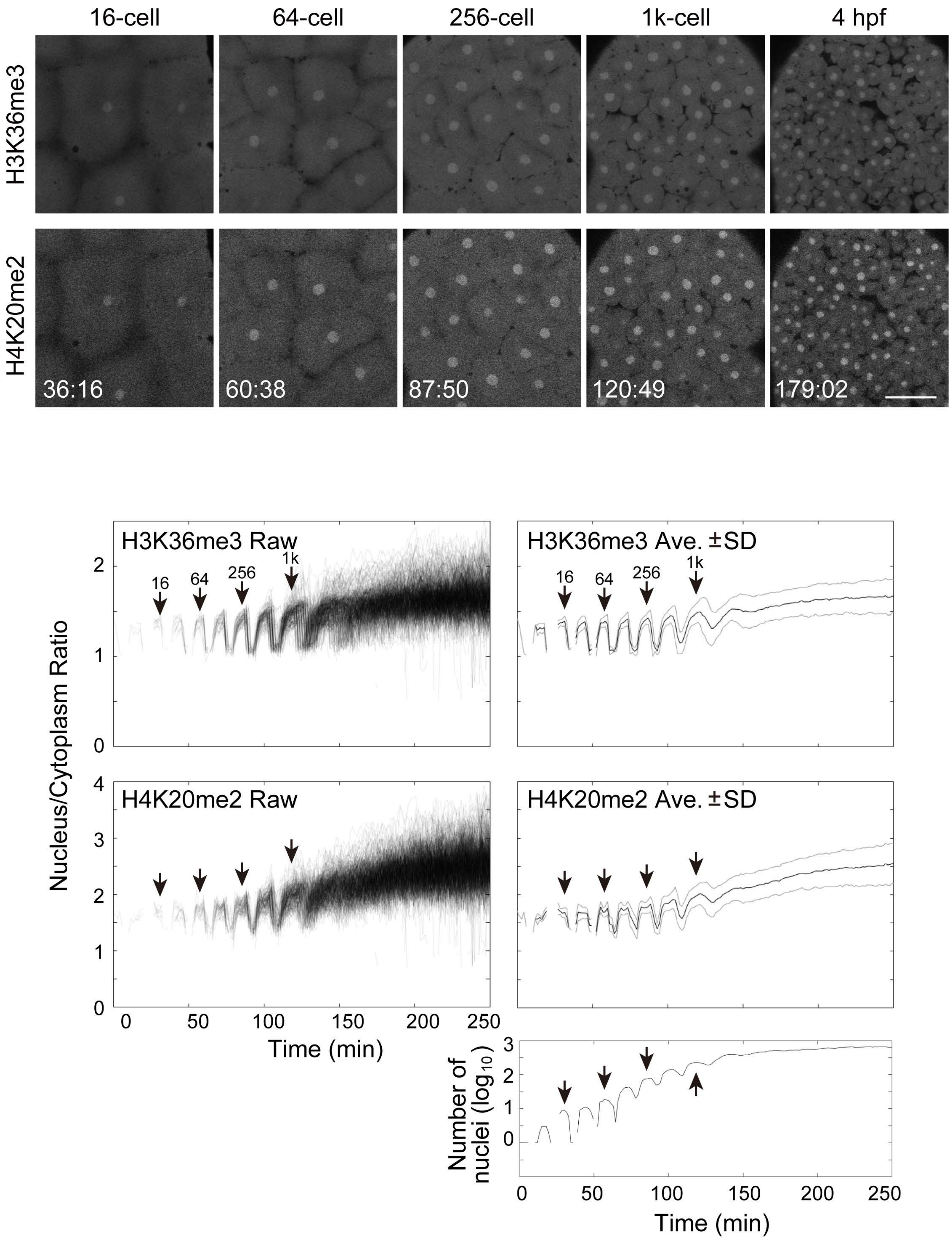

Development • Supplementary information

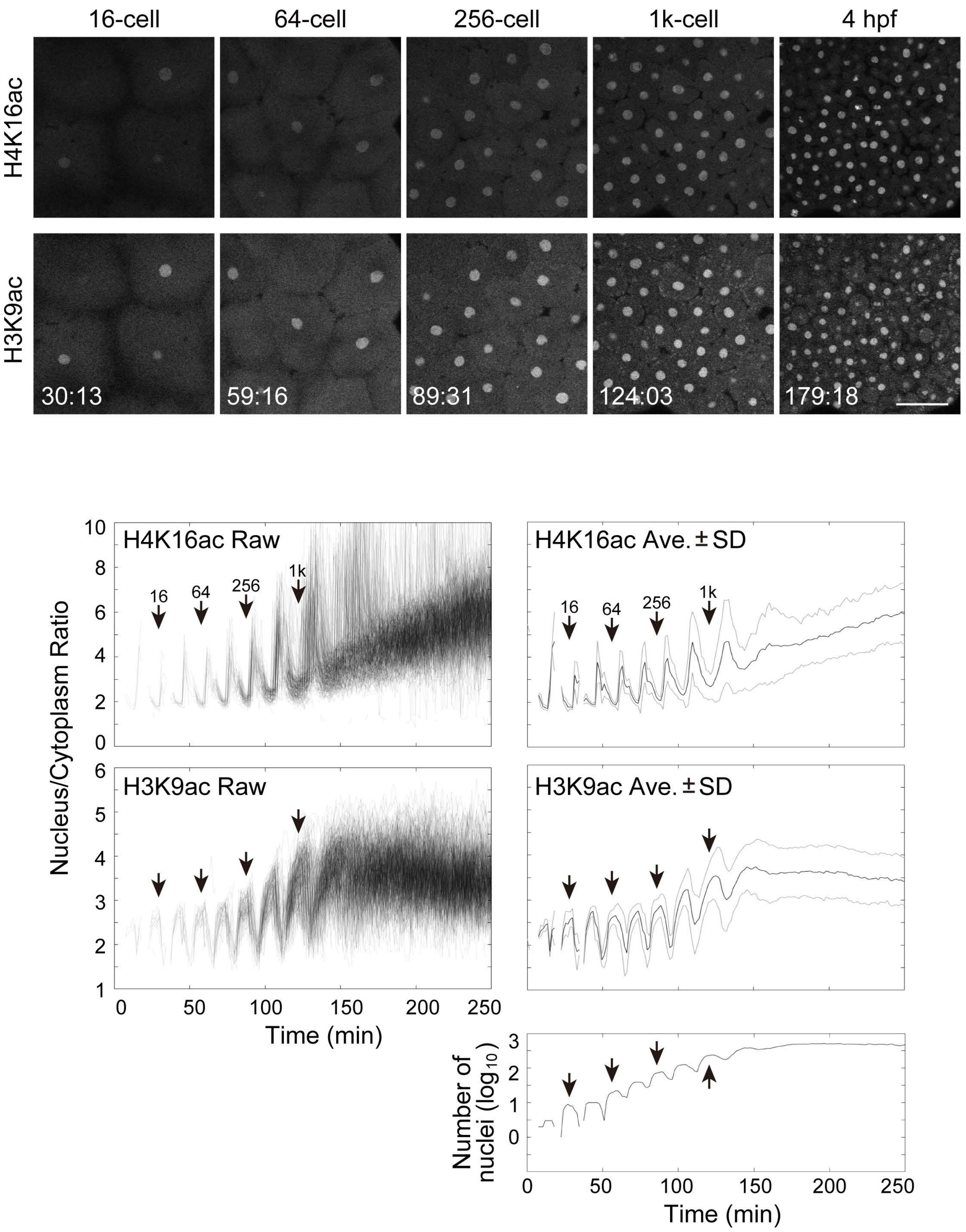

Development • Supplementary information

Figure S4. Nucleus/Cytoplasm intensity ratios of Fabs specific to various RNA polymerase II and histone modifications.

Embryos were injected with Fabs specific to various modifications (Alexa488 or Cy3) with H3K9ac (Cy5). Example images with N/C ratios of indicated Fabs (left, individual nuclei; right, averages with standard deviations; top, Alexa488; middle, Cy3) from a single embryo are shown, with the number of measured nuclei (bottom). The graphs of H3K9ac (Cy5) are also shown with H4K16ac (Alexa488). Scale bars, 100 µm.

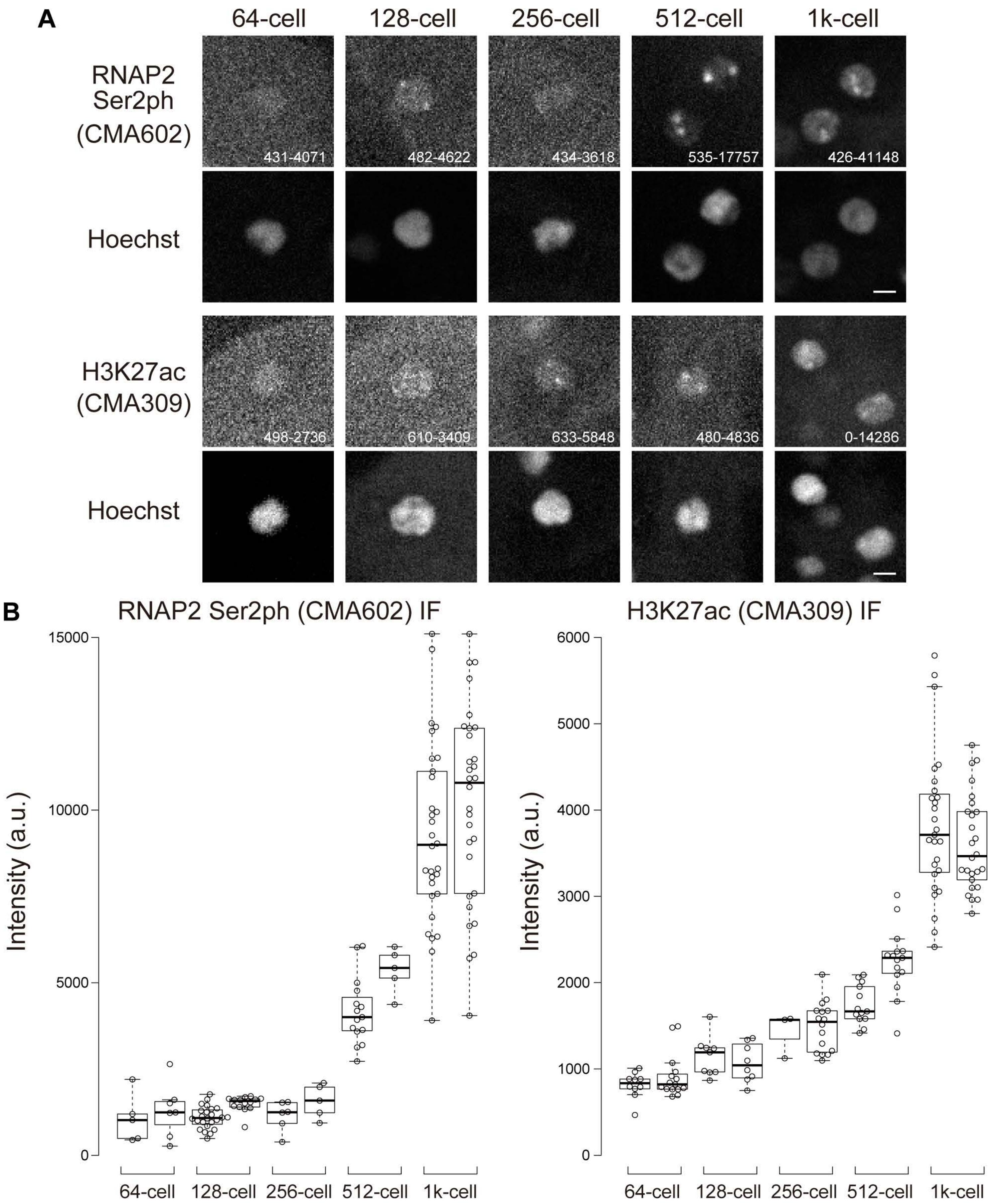
A
64-cell
128-cell
256-cell
512-cell
1k-cell
RNAP2 Ser2ph (CMA602)
431-4071
482-4622
434-3618
535-17757
426-41148
Hoechst
H3K27ac (CMA309)
498-2736
610-3409
633-5848
480-4836
0-14286
Hoechst
B
RNAP2 Ser2ph (CMA602) IF
Intensity (a.u.)
15000
10000
5000
0
64-cell
128-cell
256-cell
512-cell
1k-cell
H3K27ac (CMA309) IF
Intensity (a.u.)
6000
5000
4000
3000
2000
1000
0
64-cell
128-cell
256-cell
512-cell
1k-cell

Figure S5. Levels of RNAP2 Ser2ph and H3K27ac in zebrafish embryos at the different stages by immunofluorescence using in-house antibodies.

Embryos were fixed at the 64-, 128-, 256-, 512-, and 1k-cell stages and stained with antibodies specific to RNAP2 Ser2ph (CMA602) and H3K27ac (CMA309). (A) Representative images of nuclei. After staining with the antibodies, fluorescence images were collected using the same setting of a confocal microscope (CSU-W1) for the all stages. The contrast of each image was manually adjusted for display purposes, and their ranges in 16-bit depth are indicated. Scale bars, 10 µm. The size of foci appears larger than that in living embryos, which might be explained by the following possibilities. The focus edge might be obscured due to the background fluorescence from diffusible fraction of Fab in living cells, and/or the chromatin structure may be altered during the fixation process in fixed cells. (B) Fluorescence intensities of nuclei in single embryos were measured and boxplots were generated using BoxPlotR (http://shiny.chemgrid.org/boxplotr/). Data from two embryos at the same stage are shown. Center lines show the medians; box limits indicate the 25th and 75th percentiles as determined by R software; whiskers extend 1.5 times the interquartile range from the 25th and 75th percentiles.

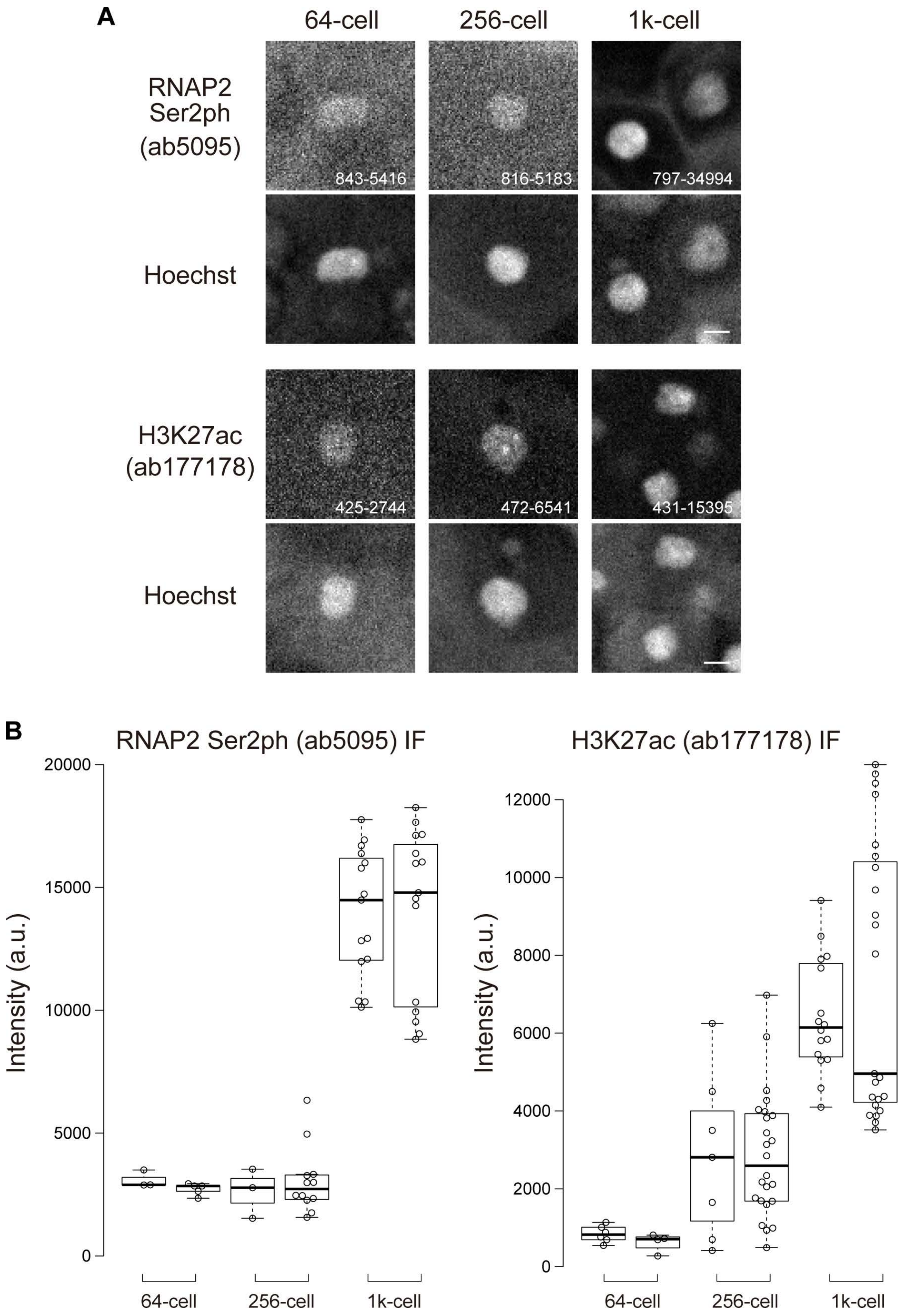
A
64-cell
256-cell
1k-cell
RNAP2 Ser2ph (ab5095)
843-5416
816-5183
797-34994
Hoechst
H3K27ac (ab177178)
425-2744
472-6541
431-15395
Hoechst
B
RNAP2 Ser2ph (ab5095) IF
H3K27ac (ab177178) IF
Intensity (a.u.)
20000
15000
10000
5000
0
12000
10000
8000
6000
4000
2000
0
64-cell
256-cell
1k-cell

Figure S6. Levels of RNAP2 Ser2ph and H3K27ac by immunofluorescence using commercial antibodies.

Embryos were fixed at the 64-, 256-, 1k-cell stages and stained with antibodies specific to RNAP2 Ser2ph (Abcam; ab5095) or H3K27ac (Abcam; a177178). (A) Representative images of nuclei. After staining with the antibodies, fluorescence images were collected using the same setting of a confocal microscope (CSU-W1) for the all stages. The contrast of each image was manually adjusted for display purposes, and their ranges in 16-bit depth are indicated. Scale bars, 10 μm. (B) Fluorescence intensities of nuclei were measured from images and boxplots were generated using BoxPlotR, as in Supplementary Fig. 5.

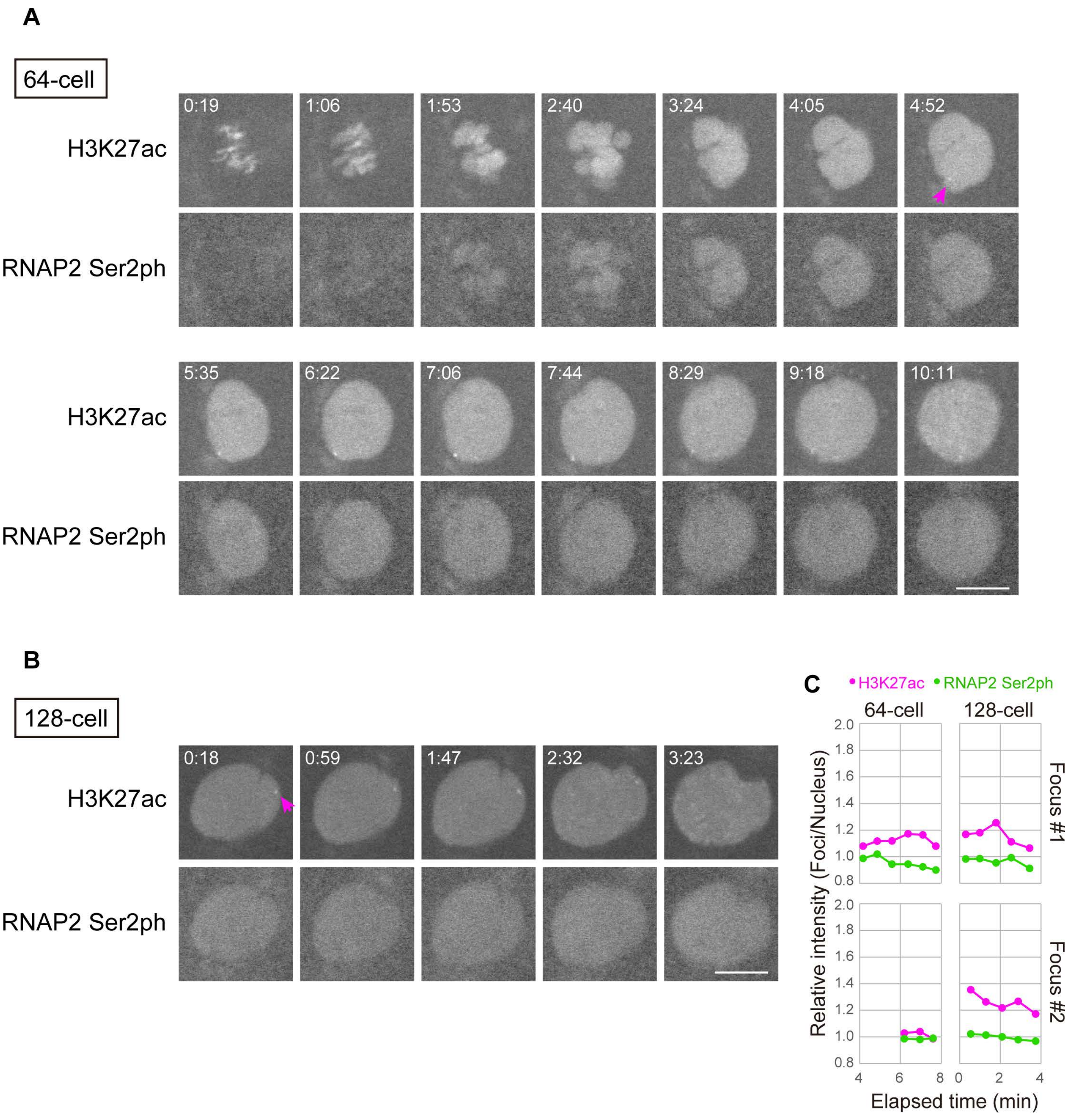

Figure S7. H3K27ac foci in the early stage embryos.
Embryos were injected with Fabs specific to RNAP2 Ser2ph (Alexa488), H3K27ac (Cy3), and H3K9ac (Cy5). (A and B) Time lapse images. Elapsed times (min:sec) are indicated. See also Supplementary Movie 4 and 5 for the 64- and 128-stages, respectively. Scale bar, 10 µm. (A) Distribution of RNAP2 Ser2ph and H3K27ac in the 64-cell stage nucleus. H3K27ac focus appeared at 4:52 (indicated by an arrow) and persisted throughout the interphase. (B) Distribution of RNAP2 Ser2ph and H3K27ac in the 128-cell stage nucleus. H3K27ac focus at 0:18 (indicated by an arrow) persisted throughout the interphase. (C) Relative enrichments of RNAP2 Ser2ph and H3K27ac on a focus over the nucleus (Foci/Nucleus). Two foci in the single nucleus were measured.

Development • Supplementary information

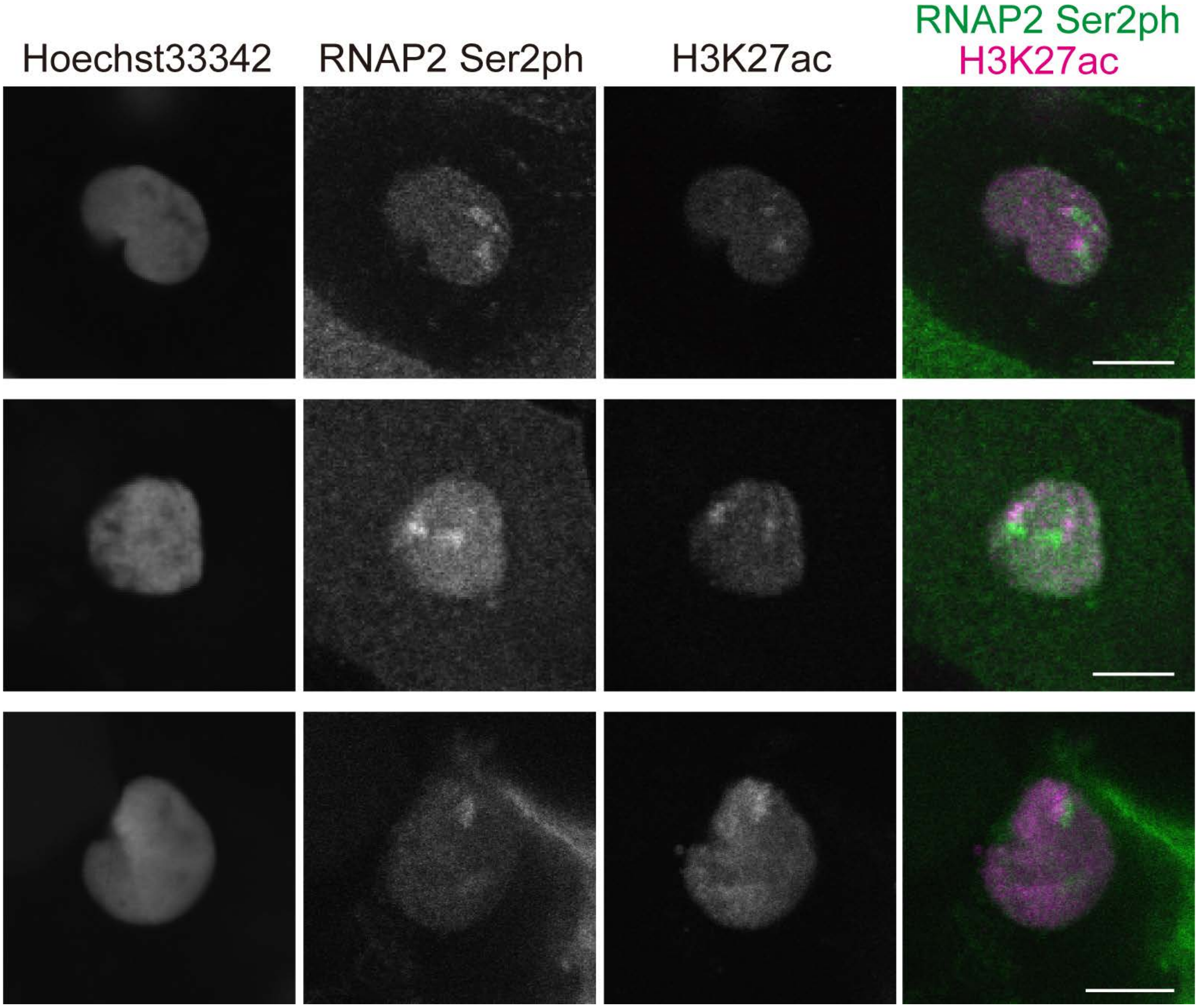

Figure S8. Double staining of the 512-stage embryos with H3K27ac and RNAP2 Ser2ph. The embryos injected with RNAP2 Ser2ph-Fab (Alexa488) were fixed at the 512-cell stage and stained with anti-H3K27ac antibody (CMA309; Cy3) and Hoechst33342. Single confocal sections with merged images are shown. RNAP2 Ser2ph and H3K27ac were observed close together. Scale bars, 10 μm.

Development • Supplementary information

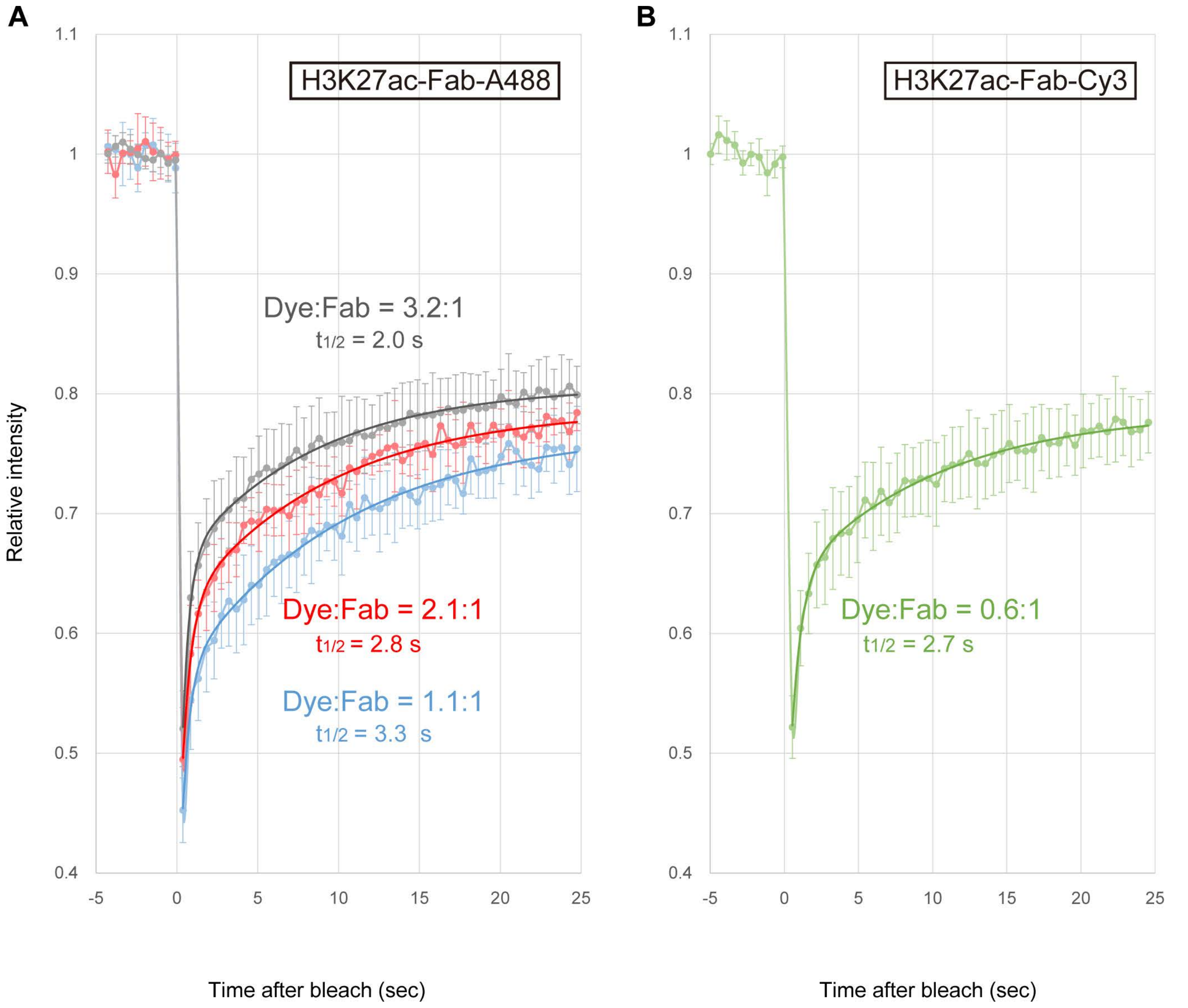

Figure S9. Fluorescence recovery after photobleaching of H3K27ac-Fab in HeLa cell nuclei. (A) Alexa Fluor 488-labeled H3K27ac-Fabs with three different dye conjugation ratios were loaded into HeLa cells and photobleaching assay was performed (gray, Dye:Fab = 3.2:1; red, 2.1:1; blue; 1.1:1). Averages ± standard deviations of 20 cells are shown. Curves after fitting to double exponential kinetics are overlaid. The half-recovery periods (t1/2) are indicated. (B) The recovery of Cy3-labeled H3K27ac-Fab (Dye:Fab = 0.6:1) is indicated, as in (A).

Development • Supplementary information

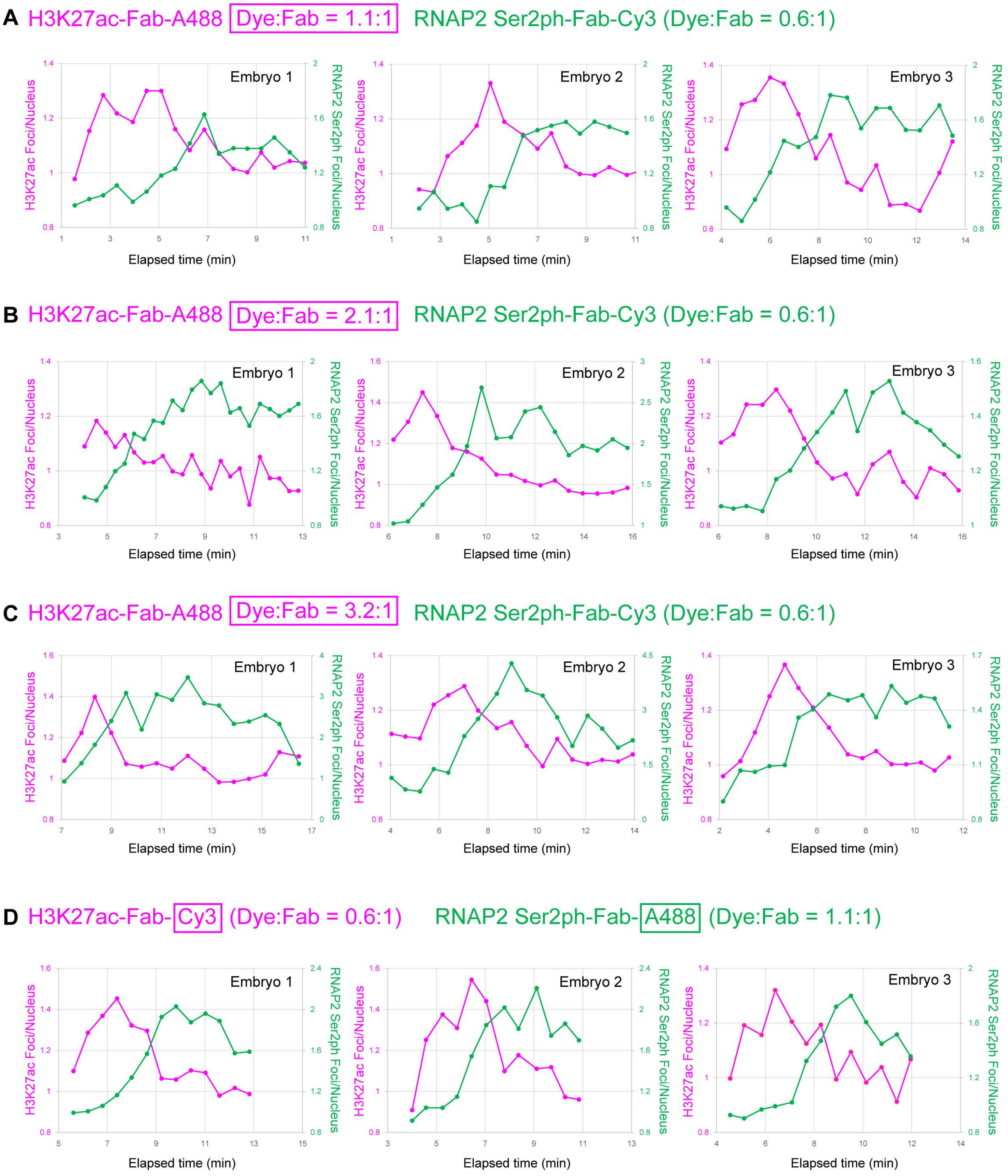

Development • Supplementary information

Figure S10. Enrichments of Fabs specific to RNAP2 Ser2ph and H3K27ac, labeled at different Dye:Fab ratios, in foci at the 1k-cell stage.
Embryos were injected with Fabs specific to RNAP Ser2ph, H3K27ac, and H3K9ac (Cy5). During the 1k-cell stage, enrichments of H3K27ac and RNAP2 Ser2ph on a single focus over the nucleus (Foci/Nuc) were measured in three different embryos. (A-C) H3K27ac-Fab labeled with Alexa488 at Dye:Fab ratio 1.1:1 (A), 2.1:1 (B), or 3.2:1 (C) was injected with RNAP2 Ser2ph-Fab (labeled with Cy3 at Dye:Fab ratio 0.6:1) and H3K9ac-Fab (Cy5). (D) H3K27ac-Fab labeled with Cy3 at Dye:Fab ratio 0.6:1 was injected with RNAP2 Ser2ph-Fab (labeled with Alexa488 at Dye:Fab ratio 1.1:1) and H3K9ac-Fab (Cy5). In all samples, H3K27ac peaked before RNAP2 Ser2ph.

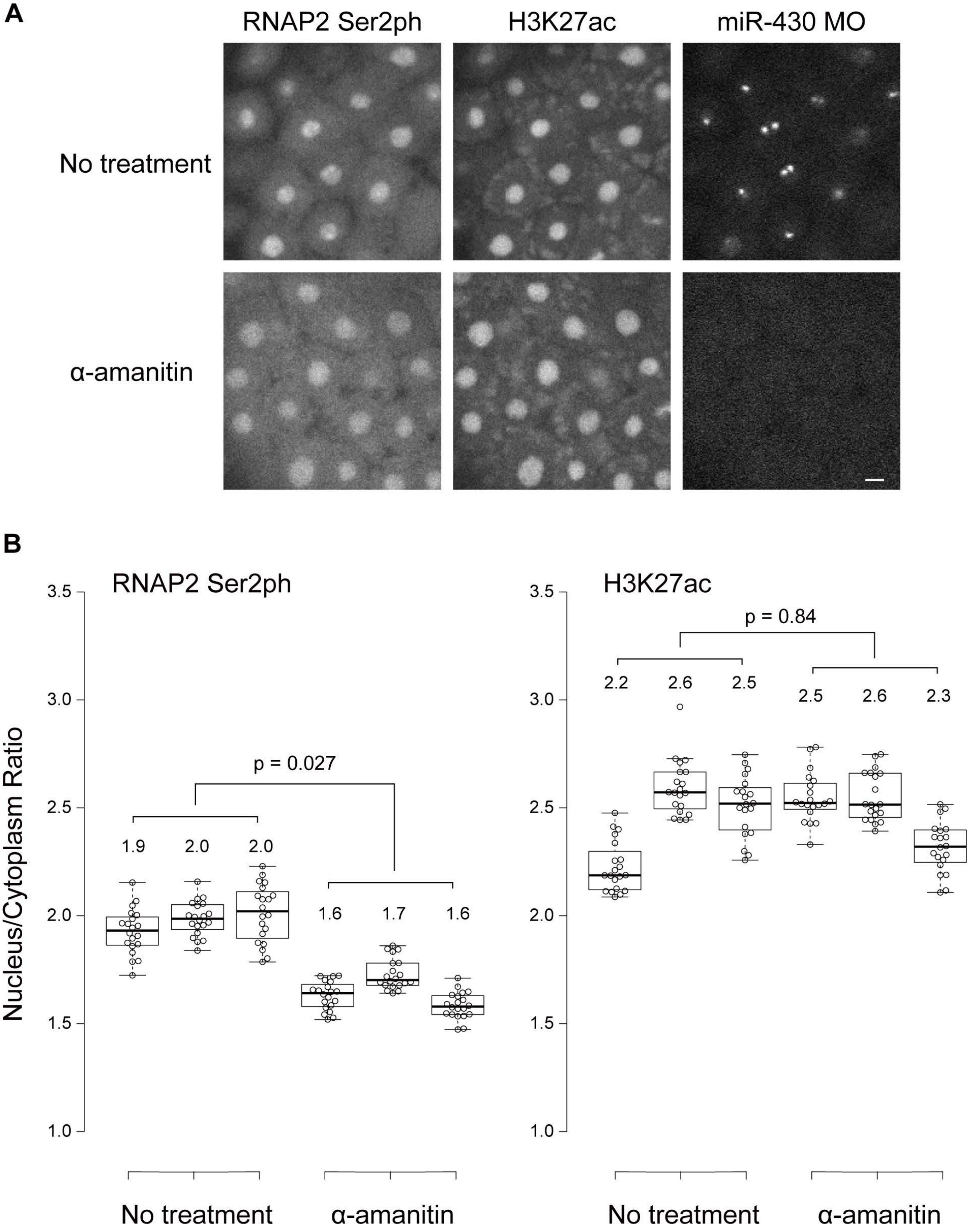
A
RNAP2 Ser2ph
H3K27ac
miR-430 MO
No treatment
α-amanitin
B
RNAP2 Ser2ph
H3K27ac
Nucleus/Cytoplasm Ratio
3.5
3.0
2.5
2.0
1.5
1.0
p = 0.027
1.9
2.0
2.0
1.6
1.7
1.6
p = 0.84
2.2
2.6
2.5
2.5
2.6
2.3
No treatment
α-amanitin

Figure S11. Effects of α-amanitin on the distribution of Fabs specific to RNAP2 Ser2ph and H3K27ac.

Embryos were injected with Fabs specific to RNAP2 Ser2ph (Alexa488) and H3K27ac (Cy3), miR-430 Morpholino (Cy5). α-amanitin was then injected (~0.25 ng in ~0.5 nl water) into some embryos, before imaging at 4-hour post fertilization (hpf). (A) Single confocal sections of embryos without (No treatment) or with injection (α-amanitin). Scale bar, 10 µm. (B) Nucleus/Cytoplasm ratios of RNAP2 Ser2ph and H3K27ac. Intensities of the nucleus and cytoplasm were measured from three different embryos, and the N/C ratios were boxplotted using BoxPlotR (http://shiny.chemgrid.org/boxplotr/). Center lines show the medians; box limits indicate the 25th and 75th percentiles as determined by R software; whiskers extend 1.5 times the interquartile range from the 25th and 75th percentiles. For statistical analysis, unpaired two-tailed Students' t-test was used with the indicated averages for individual embryos (n = 3).

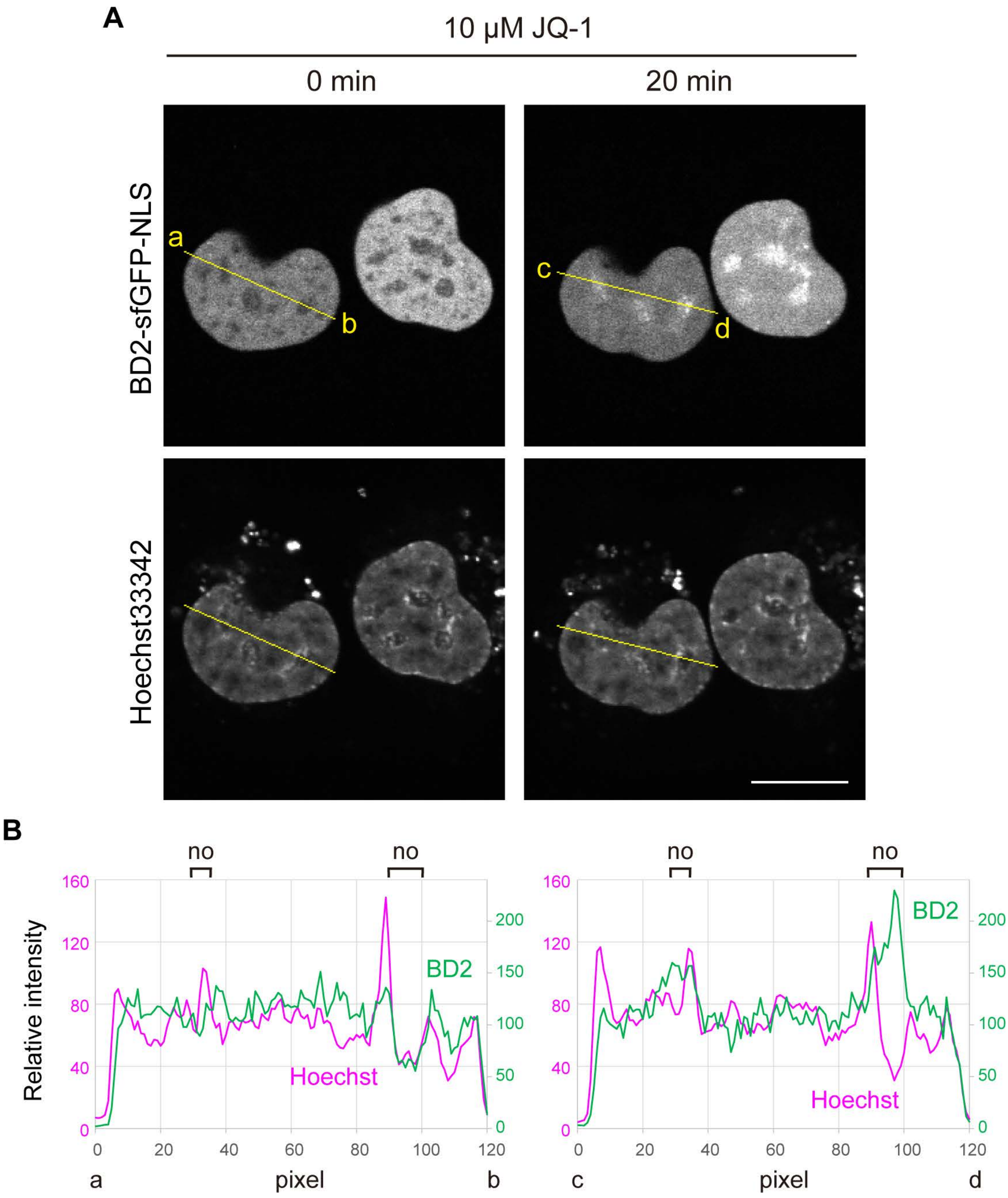

Development • Supplementary information

Figure S12. Evaluation of the bromodomain probe in U2OS cells.

To validate the effect of JQ-1, an acetyl-histone binding probe (BD2-sfGFP-NLS) was generated by fusing the bromodomain 2 from human Brd4 with super-folder GFP (sfGFP) and zebrafish nucleoplasmin nuclear localization signal. The BD2-sfGFP-NLS was expressed in human U2OS cells, which were then incubated in 0.1 μg/mL Hoechst33342, to visualize DNA, and 0.1 μM trichostatin A, a histone deacetylase inhibitor, to facilitate the acetylation level, for 4 h. (A) Single confocal sections collected before (0 min) and 20 min after the addition of 10 μM JQ-1. Scale bar, 10 μm. (B) Line intensity profiles. The intensities of BD2-sfGFP-NLS (BD2, green) and Hoechst33342 (magenta) are plotted along lines from a to b (0 min) and c to d (20 min in JQ1). The area of nucleoli are indicated as "no". The BD2-sfGFP-NLS showed less enrichment in Hoechst-dense heterochromatin and in nucleoli without JQ-1, suggesting its euchromatin binding. In the presence of JQ-1, the BD2-sfGFP-NLS distributed more homogenously in the nucleoplasm and concentrated in nucleoli, probably because JQ-1 blocked its chromatin binding.

Development • Supplementary information

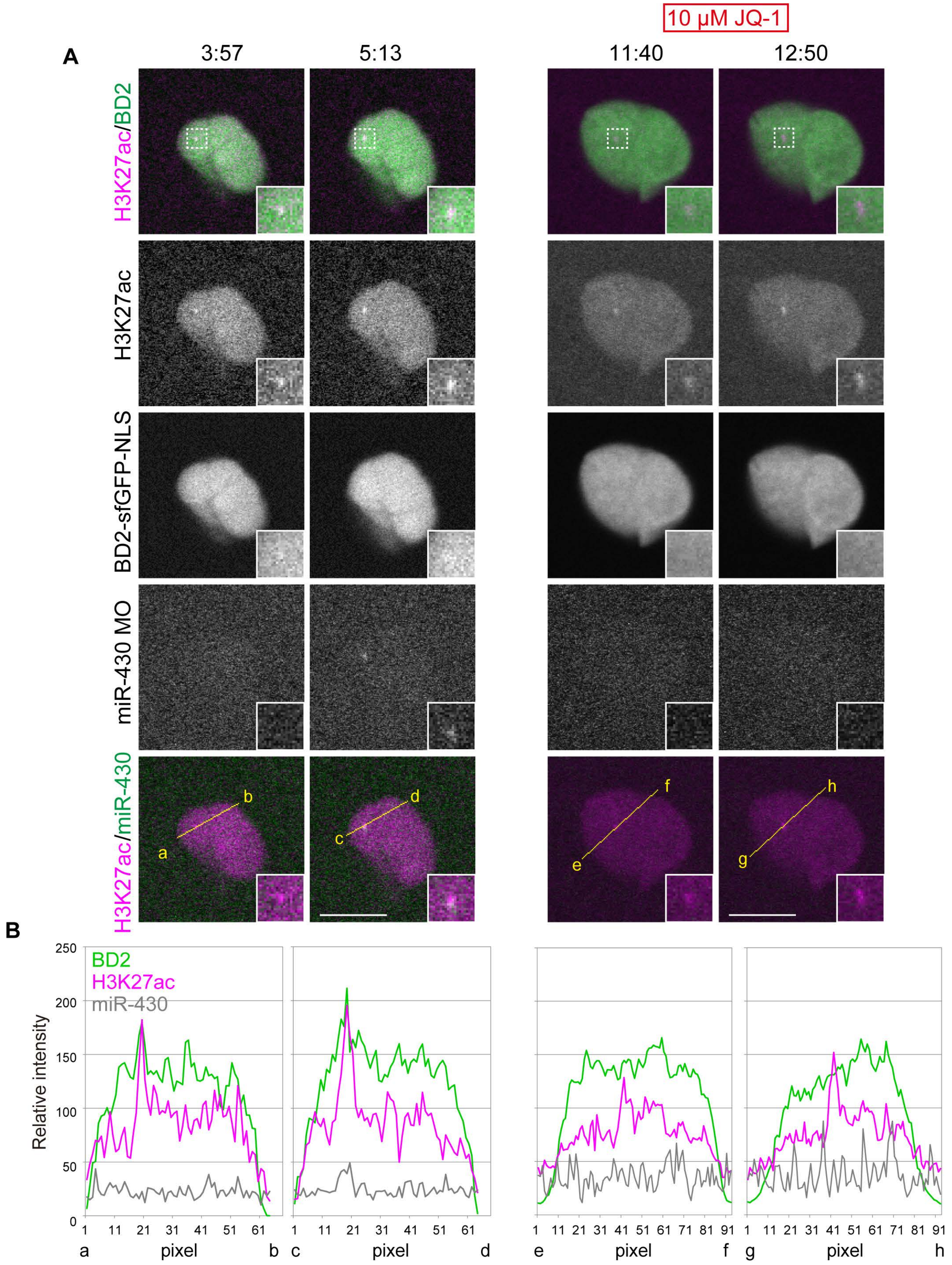

Development • Supplementary information

Figure S13. Validation of the effect of JQ-1 in zebrafish embryos.
Embryos were injected with mRNA encoding the BD2-sfGFP-NLS with H3K27ac-Fab (Cy3) and miR-430 Morpholino (Cy5). The embryos without or with 10 μM JQ-1 were imaged at the 1k-cell stage. (A) Single confocal images at two time points. Insets show magnified views of the indicated area containing H3K27ac foci. The elapsed time (min:sec) is indicated above. Scale bar, 10 μm. (B) Line intensity profiles. The intensities of BD2-sfGFP-NLS (BD2, green), H3K27ac (magenta), and miR-430 (grey) are plotted along lines a-b, c-d, e-f, and g-h. The BD2-sfGFP-NLS showed subtle concentrations on H3K27ac focus without JQ-1, but such concentrations and miR-430 transcripts were not detected in the presence of JQ-1.

Development • Supplementary information

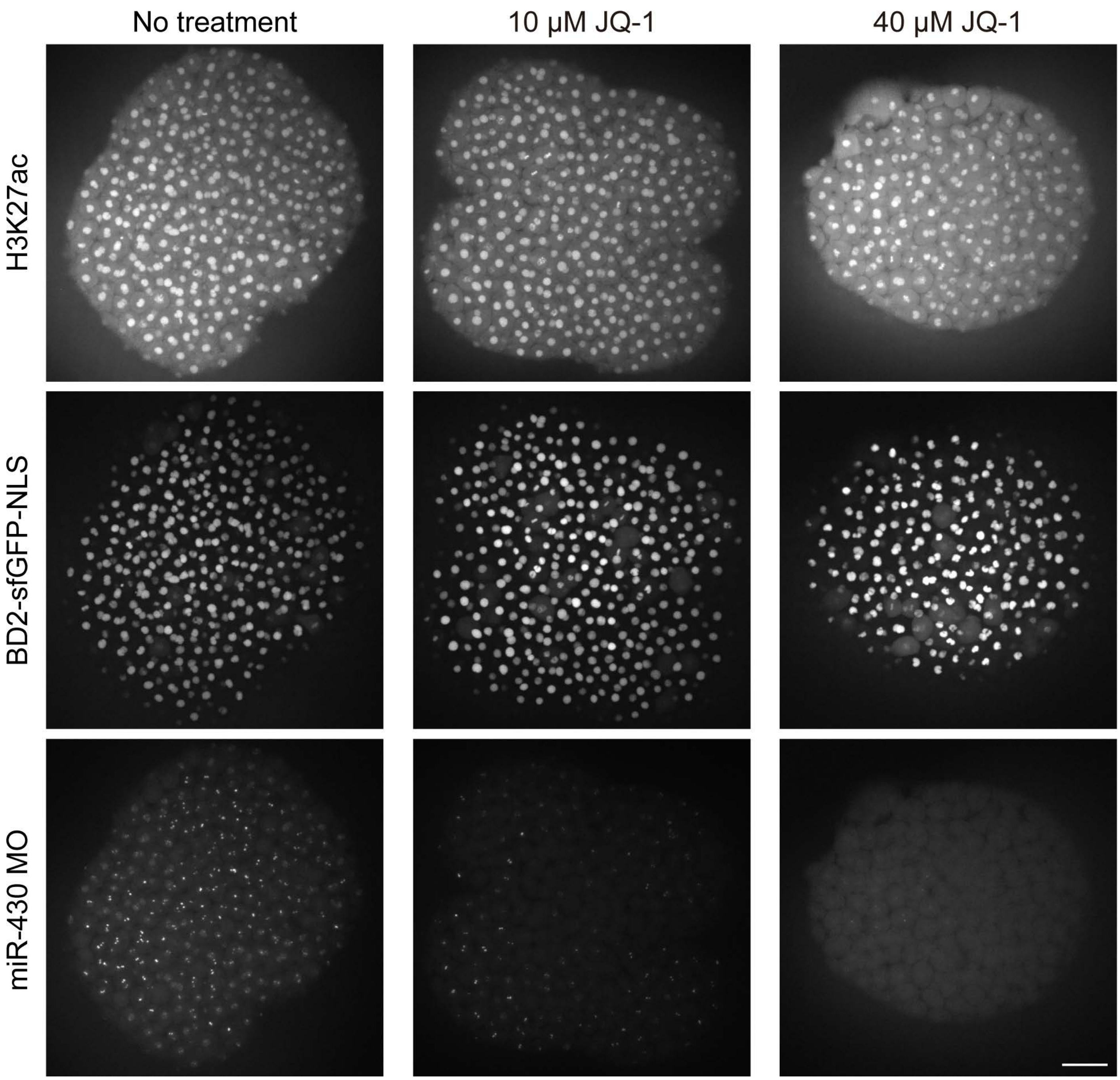

Figure S14. Effects of JQ-1 on miR-430 transcription in zebrafish embryos. Embryos were injected with mRNA encoding the BD2-sfGFP-NLS, H3K27ac-Fab (Cy3), and miR-430 Morpholino (Cy5), and incubated in 0, 10, and 40 μM JQ-1, and imaged at 4 hpf. miR-430 transcripts were detected less with variations depending on nuclei in 10 μM and scarcely detected in all nuclei in 40 μM JQ-1. Scale bar, 100 μm.

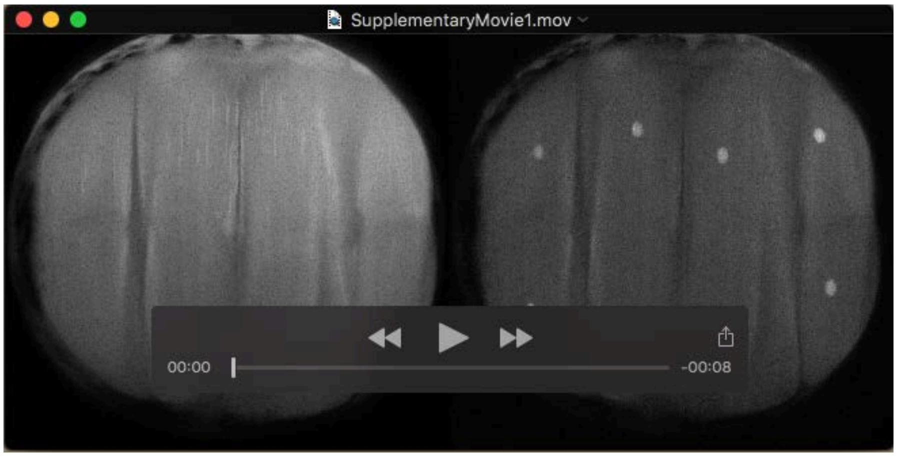

Movie 1. Live-imaging of RNAP2 Ser2ph and H3K9ac in zebrafish developing embryo.
The embryo injected with Fabs specifics to RNAP2 Ser2ph (Alexa488) and H3K9ac (Cy5) was imaged every minute from the 8-cell stage using a light sheet microscope (SiMView). The maximum intensity projections of 198 z-sections with 2 μm intervals are shown.

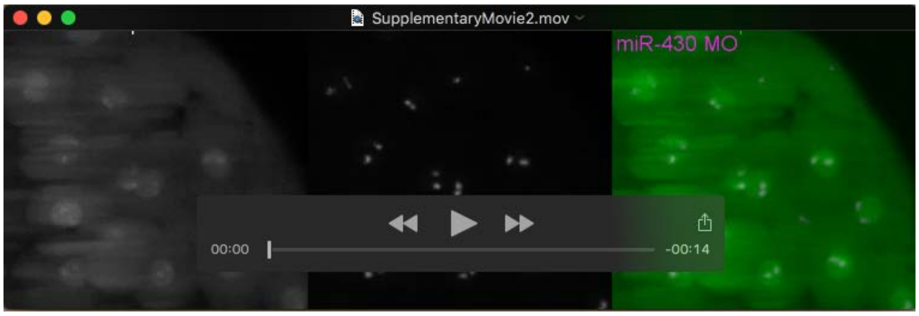

Movie 2. Live-imaging of RNAP2 Ser2ph and miR-430 transcripts in the 1k- to 2k-cell stage embryo.
The embryo injected with RNAP2 Ser2ph-Fab (Alexa488) and miR-430 Morpholino probe (Cy3) was imaged every minutes using a light sheet microscope (SiMView). The maximum intensity projections of 30 z-sections with 2 μm intervals with merged images are shown.

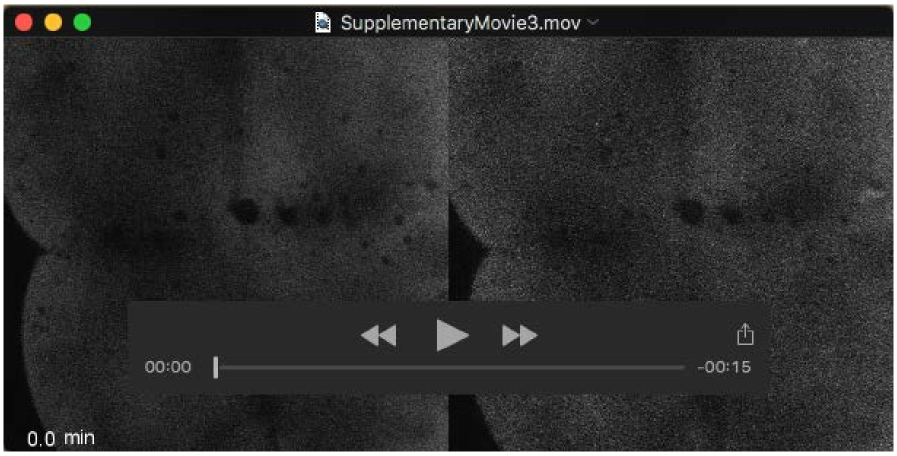

Movie 3. Live-imaging of RNAP2 Ser2ph and H3K27ac in zebrafish developing embryo.
The embryo injected with Fabs specific to RNAP2 Ser2ph (Alexa488), H3K27ac (Cy3), and H3K9ac (Cy5) was imaged using a confocal microscope (FV1000). Twenty five z-sections with 4 µm intervals were collected, and single confocal sections for RNAP2 Ser2ph and H3K27ac were selected to make a movie. Elapsed time is indicated as min:sec.

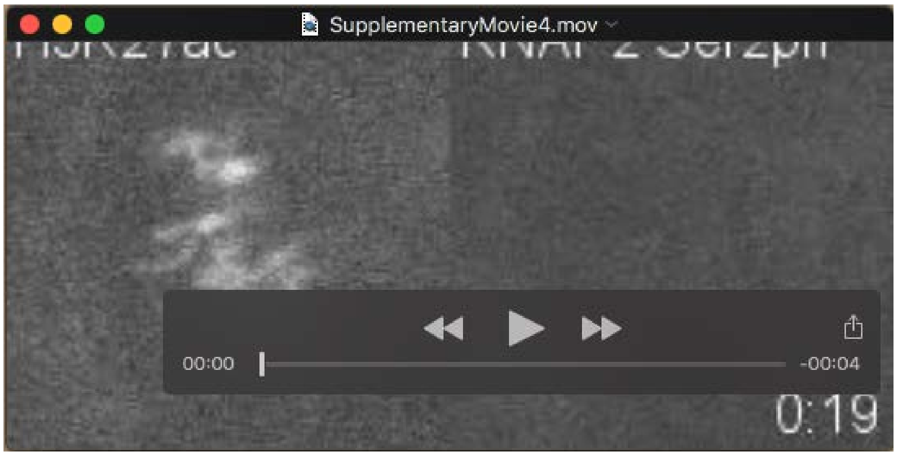

Movie 4. Live-imaging of RNAP2 Ser2ph and H3K27ac in a single nucleus at the 64-cell stage.
An embryo was injected with Fabs specific to RNAP2 Ser2ph (Alexa488), H3K27ac (Cy3), and H3K9ac (Cy5) were imaged using a confocal microscope (FV1000). Sixteen z-sections with 1 µm intervals were collected, and single sections for RNAP2 Ser2ph and H3K27ac were selected to make a movie for the 64-cell stage. Elapsed time is indicated as min:sec.

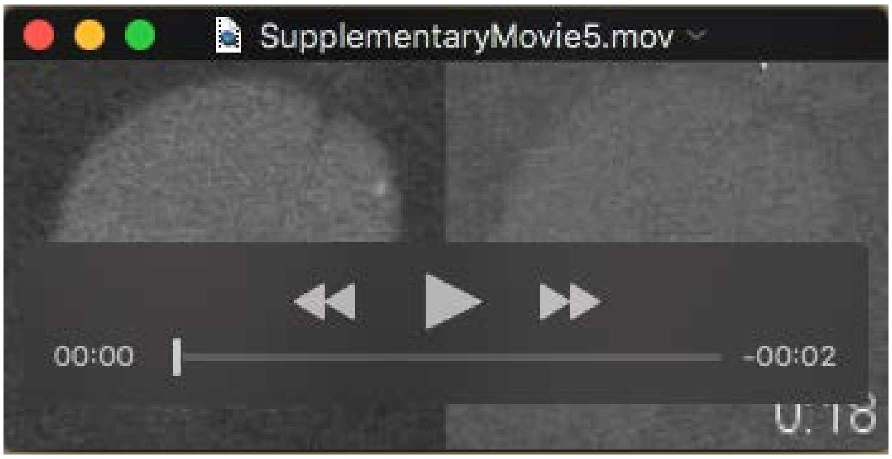

Movie 5. Live-imaging of RNAP2 Ser2ph and H3K27ac in a single nucleus at the 128-cell stage.

An embryo was injected with Fabs specific to RNAP2 Ser2ph (Alexa488), H3K27ac (Cy3), and H3K9ac (Cy5) were imaged using a confocal microscope (FV1000). Fifteen z-sections with 1 µm intervals were collected, and single sections for RNAP2 Ser2ph and H3K27ac were selected to make a movie for the 128-cell stage. Elapsed time is indicated as min:sec.

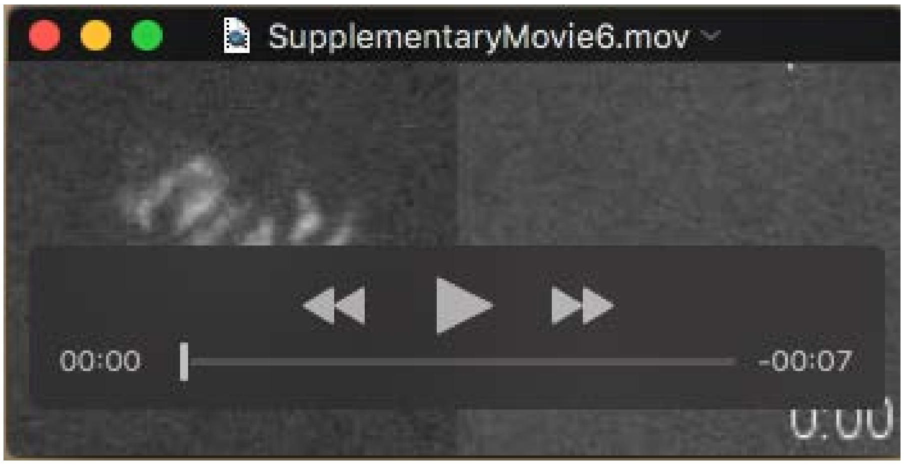

Movie 6. Live-imaging of RNAP2 Ser2ph and H3K27ac in a single nucleus at the 256-cell stage.

An embryo was injected with Fabs specific to RNAP2 Ser2ph (Alexa488), H3K27ac (Cy3), and H3K9ac (Cy5) were imaged using a confocal microscope (FV1000). Thirteen z-sections with 1 µm intervals were collected, and single sections for RNAP2 Ser2ph and H3K27ac were selected to make a movie for the 256-cell stage. Elapsed time is indicated as min:sec.

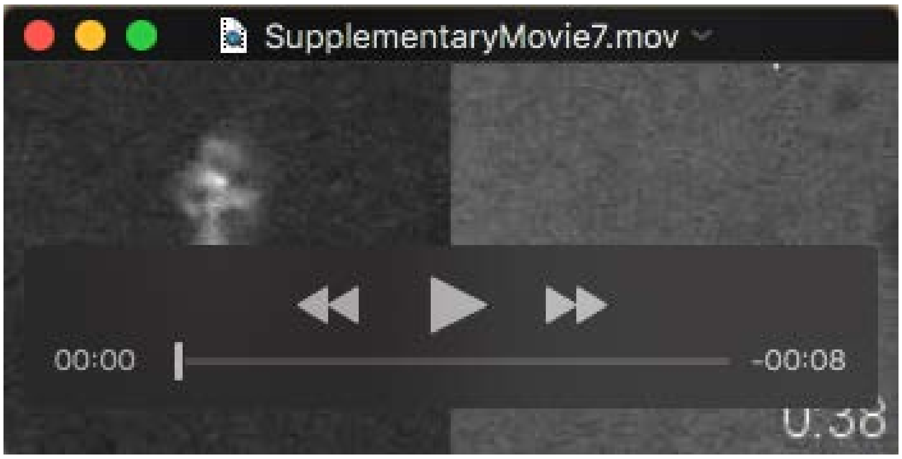

Movie 7. Live-imaging of RNAP2 Ser2ph and H3K27ac in a single nucleus at the 512-cell stage.
An embryo was injected with Fabs specific to RNAP2 Ser2ph (Alexa488), H3K27ac (Cy3), and H3K9ac (Cy5) were imaged using a confocal microscope (FV1000). Sixteen z-sections with 1 µm intervals were collected, and single sections for RNAP2 Ser2ph and H3K27ac were selected to make a movie for the 512-cell stage. Elapsed time is indicated as min:sec.

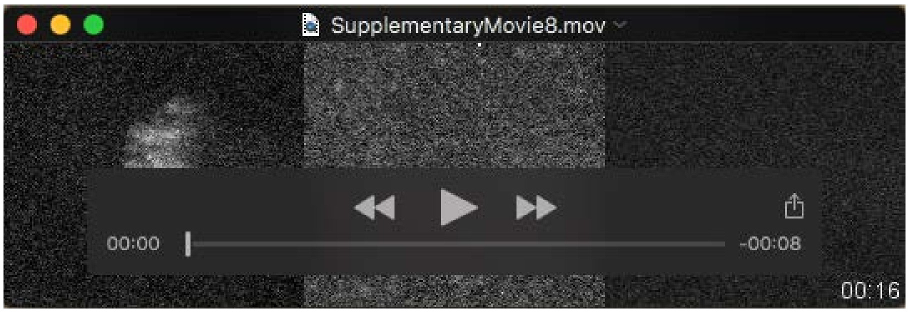

Movie 8. Live-imaging of RNAP2 Ser2ph, H3K27ac, and miR-430 transcripts in a single nucleus at the 512-cell stage.
An embryo was injected with Fabs specific for RNAP2 Ser2ph (Cy3) and H3K27ac (Alexa488), and miR-430 Morpholino (Cy5), and imaged using a confocal microscope (FV1000). Twenty z-sections with 1 µm intervals were collected, and single sections were selected to make a movie for the 512-cell stage. Elapsed time is indicated as min:sec.

Development • Supplementary information

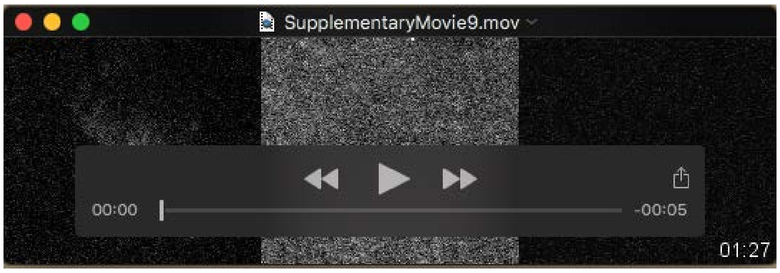

Movie 9. Live-imaging of RNAP2 Ser2ph, H3K27ac, and miR-430 transcripts in a single nucleus at the 512-cell stage in the presence of α-amanitin.
An embryo was injected with Fabs specific for RNAP2 Ser2ph (Cy3) and H3K27ac (Alexa488), and miR-430 Morpholino (Cy5), and then with α-amanitin, before imaging using a confocal microscope (FV1000). Twenty z-sections with 1 μm intervals were collected, and single sections were selected to make a movie for the 512-cell stage. Elapsed time is indicated as min:sec.

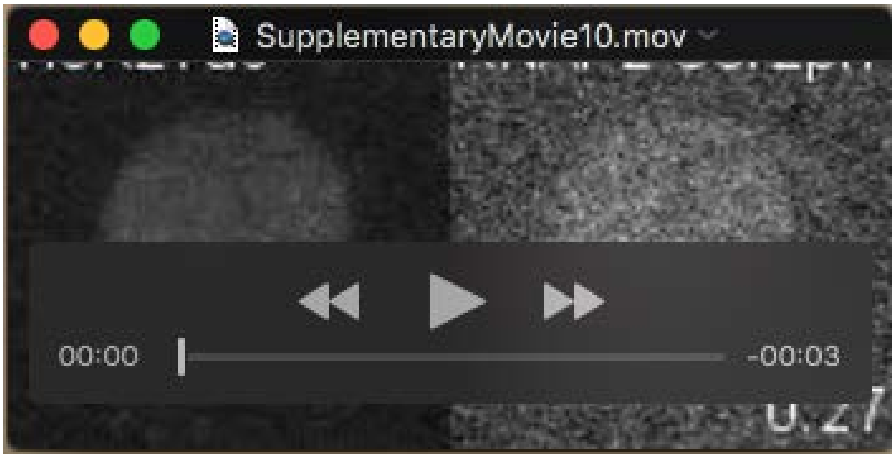

Movie 10. Live-imaging of RNAP2 Ser2ph and H3K27ac in a single nucleus at the 256-cell stage in the presence of 10 μM JQ-1.
An embryo was injected with Fabs specific to RNAP2 Ser2ph (Alexa488), H3K27ac-Fab (Cy3), H3K9ac (Cy5) and soaked in 10 μM JQ-1, before and during imaging using a confocal microscope (FV1000). Twenty z-sections with 1 μm intervals were collected, and single sections were selected to make a movie for the 256-cell stage. Elapsed time is indicated as min:sec.

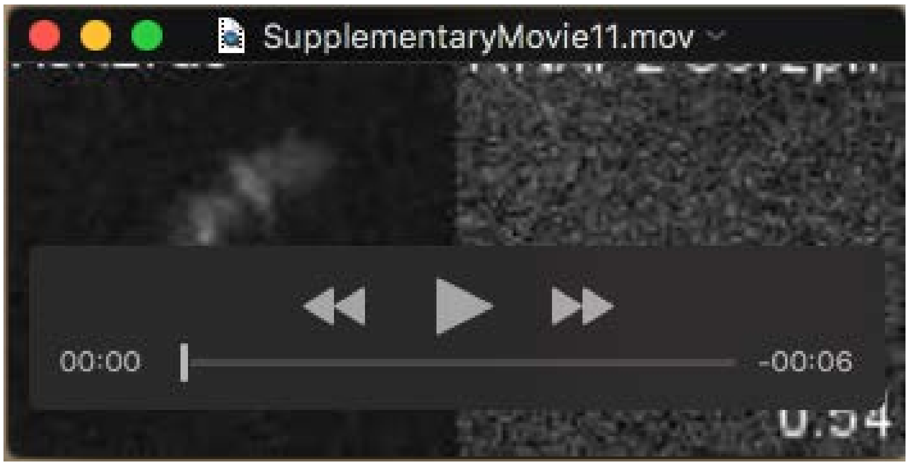

Movie 11. Live-imaging of RNAP2 Ser2ph and H3K27ac in a single nucleus under the presence of 10 μM JQ-1 at 512-cell stage.

An embryo was injected with Fabs specific to RNAP2 Ser2ph (Alexa488), H3K27ac-Fab (Cy3), and H3K9ac (Cy5) and soaked in 10 μM JQ-1, before and during imaging using a confocal microscope (FV1000). Twenty z-sections with 1 μm intervals were collected, and single sections were selected to make a movie for the 512-cell stage. Elapsed time is indicated as min:sec.
